# *Polygonatum cyrtonema* polysaccharides reshape the gut microbiota to ameliorate dextran sodium sulfate-induced ulcerative colitis in mice

**DOI:** 10.3389/fphar.2024.1424328

**Published:** 2024-06-05

**Authors:** Chaoyou Lin, Dawei Song, Shangwen Wang, Yunfei Chu, Changxing Chi, Sining Jia, Mengyi Lin, Chenbei He, Chengxi Jiang, Fanghua Gong, Qiongzhen Chen

**Affiliations:** ^1^ School of Life and Environmental Sciences, Wenzhou University, Wenzhou, China; ^2^ Mount Jiuhuashan Sealwort Research Institute, Chizhou, China; ^3^ School of Pharmacy, Wenzhou Medical University, Wenzhou, China; ^4^ China Department of Endocrinology, Yanbian University Hospital, Yanji, China

**Keywords:** *Polygonatum cyrtonema* polysaccharides, ulcerative colitis, inflammation, oxidative stress, mucosal damage, gut microbiota

## Abstract

Ulcerative colitis (UC) is a chronic inflammatory bowel disease characterized inflammatory imbalance, intestinal epithelial mucosal damage, and dysbiosis of the gut microbiota. *Polygonatum cyrtonema* polysaccharides (PCPs) can regulate gut microbiota and inflammation. Here, the different doses of PCPs were administered to dextran sodium sulfate-induced UC mice, and the effects of the whole PCPs were compared with those of the fractionated fractions PCP-1 (19.9 kDa) and PCP-2 (71.6 and 4.2 kDa). Additionally, an antibiotic cocktail was administered to UC mice to deplete the gut microbiota, and PCPs were subsequently administered to elucidate the potential role of the gut microbiota in these mice. The results revealed that PCP treatment significantly optimized the lost weight and shortened colon, restored the balance of inflammation, mitigated oxidative stress, and restored intestinal epithelial mucosal damage. And, the PCPs exhibited superior efficacy in ameliorating these symptoms compared with PCP-1 and PCP-2. However, depletion of the gut microbiota diminished the therapeutic effects of PCPs in UC mice. Furthermore, fecal transplantation from PCP-treated UC mice to new UC-afflicted mice produced therapeutic effects similar to PCP treatment. So, PCPs significantly ameliorated the symptoms, inflammation, oxidative stress, and intestinal mucosal damage in UC mice, and gut microbiota partially mediated these effects.

## 1 Introduction

Ulcerative colitis (UC) is primarily characterized by chronic inflammation and ulceration of the inner mucosal lining of the rectum and colon (large intestine). The disease has a recurrent and long-lasting course, and its prevalence is approximately 156–291 cases per 100,000 individuals with a progressively increasing trend (Shen X. et al., 2021; Zagorowicz et al., 2022). Geographical, age-related, genetic, and environmental factors contribute to the pathogenesis of UC. In addition, UC is also linked to excessive oxidative stress, inflammation imbalance, intestinal epithelial mucosa damage, and gut microbiota dysbiosis (Liu et al., 2021; Huang X. et al., 2023). Clinical treatment options for UC include pharmacologic and surgical approaches. Corticosteroids, 5-aminosalicylates, and immunosuppressants are the common therapeutic drugs for UC, and surgical procedures involve total proctocolectomy in patients (Baumgart and Sandborn, 2007; Niu et al., 2021). However, these therapeutic strategies induce varying degrees of physiologic damage.

In recent years, polysaccharides from natural resources such as *Gracilaria lemaneiformis* polysaccharides, *Dendrobium Officinaleon* polysaccharides and *Tremella fuciformis* polysaccharides have been reported to be potentially effective drugs for UC treatment with good therapeutic efficacy and high safety (Liang et al., 2018; Xiao et al., 2021; Lu et al., 2022). *Polygonatum cyrtonema* Hua is a medicinal and edible herbaceous plant belonging to the genus *Polygonatum* of the family *Liliaceae*. The plant has potential therapeutic and nutritional benefits, including immune-enhancing, blood glucose-lowering, lipid-lowering, antioxidant, and anti-inflammatory effects (He et al., 2021; Teng et al., 2023). *Polygonatum cyrtonema* polysaccharides (PCPs) show potent scavenging properties against free radicals and notably reduce the expression of inflammatory cytokines, such as IL-1β, IL-6, and TNF-α, in mouse embryonic fibroblasts (3T3-L1) (Li et al., 2018; Cai et al., 2019). In addition, PCPs can diminish the expression of lactic acid, blood urea nitrogen, and malondialdehyde (MDA) in mouse serum, thereby attenuating oxidative stress (Shen W.-D. et al., 2021). PCPs also modulate the composition, abundance, and diversity of gut microbiota in high-fat-diet-fed mice. They increase the abundance of gut bacteria, such as *Firmicutes* and *Lactobacilli*, while reducing the abundance of *Bacteroidetes*, thereby regulating the ecological balance of the gut microbiome (Yang et al., 2021; Luo et al., 2022).

Our findings revealed that PCPs mitigated oxidative stress in mice, optimized the balance of inflammatory factors, alleviated intestinal epithelial mucosa damage, and modulated the composition and metabolism of gut microbiota. The unfractionated PCPs showed superior efficacy in ameliorating the symptoms of UC in mice compared with the fractionated fractions. Depletion of the gut microbiota using an antibiotic cocktail (ABX) significantly diminished the therapeutic effects of PCPs. Notably, the transplantation of gut microbiota from PCP-treated UC mice into new UC-afflicted mice produced therapeutic effects similar to those observed with direct PCP treatment. Therefore, gut microbiota partially mediates the therapeutic effects of PCPs on UC.

## 2 Materials and methods

### 2.1 Plant materials and materials

Six to seven years old nine steamed and nine sunned *P. cyrtonema* was purchased from Chizhou Jiuhuafu Jinlian Wisdom Agriculture Co Ltd. (Chizhou, China). Dextran Sulfate Sodium Salt (CAS No. 9011-18-1) was purchased from Yeasen Biotechnology (Shanghai) Co., Ltd. Sulafasalazine (CAS No.599-79-1) was purchased from Sigma Aldrich Company (United States). Tight junction protein ZO-1 antibody (zonaoccludens-1, AF5145), claudin 1 protein antibody (AF0127), and α- SMA (alpha smooth muscle Action, α- SMA, AF1032) purchased from Jiangsu Qinke Biological Research Center Co., Ltd. (Jiangsu, China). Occludin antibody (66378-1-Ig), TGF-beta 1 antibody (Transforming Growth Factor beta 1, 21898-1-AP), GAPDH (glyceraldehyde-3-phosphate dehydrogenase, 60004-1-Ig), HRP-conjugated Affinipure Goat Anti-Rabbit IgG (H + L) (SA00001-2) and HRP-conjugated Affinipure Goat Anti-Mouse IgG (H + L) (SA00001-1) were purchased from Proteintech Group, Inc (China). RNA isolater Total RNA Extraction Reagent, HiScript III All-in-one RT SuperMix Perfect for qPCR(R333) and Taq Pro Universal SYBR qPCR Master Mix (Q712) were purchased from Nanjing Novozymes Bioscience and Technology Co Ltd. (Jiangsu, China). Mouse MDA ELISA KIT (JL13329), Mouse SOD ELISA KIT (JL12237) and Mouse GSH ELISA KIT (JL20360) were purchased from Shanghai Jianglai Biotechnology Co Ltd. (Shanghai, China). All other chemical reagents are analytically pure reagents.

### 2.2 Extraction of PCPs

PCPs was prepared according to the previously reported method (Xiao et al., 2021). Briefly, *P. cyrtonema* Hua slices (50 g) were weighed and boiled three times (each cycle of 2 h) with 10 × volume of water. The mixture was filtered through a 300-mesh filter cloth after each boiling cycle, and the three filtrates were combined. The combined filtrate was concentrated to 100 mL using a rotary evaporator, and anhydrous ethanol was added until the alcohol content reached 75%. The mixture was then allowed to stand overnight at 4°C, filtered, and dried at 45°C to obtain crude PCPs.

### 2.3 Purification and structural characterization of PCPs

This part of the experimental method is based on the article published by Xie et al., 2020 with slight modifications. In brief, The crude PCP sample was mixed with 8 × volume of petroleum ether, subjected to ultrasonic treatment, and allowed to stand overnight for defatting. The defatted PCP sample was then dissolved in water and stirred with 30% H_2_O_2_ for decolorization. Next, proteins were removed from the PCP solution using the Sevag method to obtain purified PCPs. DEAE–Sepharose Fast Flow resin was used for anion exchange chromatography to further purify the PCPs. The column was equilibrated, and defatted, decolored, and deproteinized PCPs were dissolved in water and subjected to a gradient elution using water and 1.5 M NaCl. Fractions were collected, concentrated, dialyzed, centrifuged, and freeze-dried to obtain PCP fraction 1 (PCP-1) and PCP fraction 2 (PCP-2). For further assessment, 10 mg of purified PCPs was hydrolyzed, derivatized, and subjected to high-performance liquid chromatography (HPLC, 1200 Infinitely, Agilent Technologies, United States) for analysis of monosaccharide composition of PCPs. The molecular weight of the polysaccharide sample (20 mg/mL) was determined using high-performance gel permeation chromatography (GPC, 1100 Series, Agilent, United States) using a TSK-gel G3000 PWXL chromatography column.Each polysaccharide sample (PCP-1 and PCP-2) was mixed with dry Potassium Bromide (1:1, w/w) separately and ground to obtain a homogeneous mixture. The resulting mixture was then pressed into transparent discs. Fourier-transform infrared (FT-IR, Nicolet iS50, Thermo, USA) spectra were obtained by scanning in the range of 4000–400 cm^−1^ using an FT-IR spectrometer.The polysaccharide sample (100 mg) was dissolved in 1 mL of ultrapure water. The UV spectra of PCP-1 and PCP-2 in the range of 200–600 nm were analyzed using a UV-visible spectrophotometer (Ultrospec 4300 pro, United States). The structure of the polysaccharides was determined using a high-resolution field-emission SEM (Hitachi regulus 8220, Hitachi, Japan).

### 2.4 Animal experiments

**FIGURE 1:**
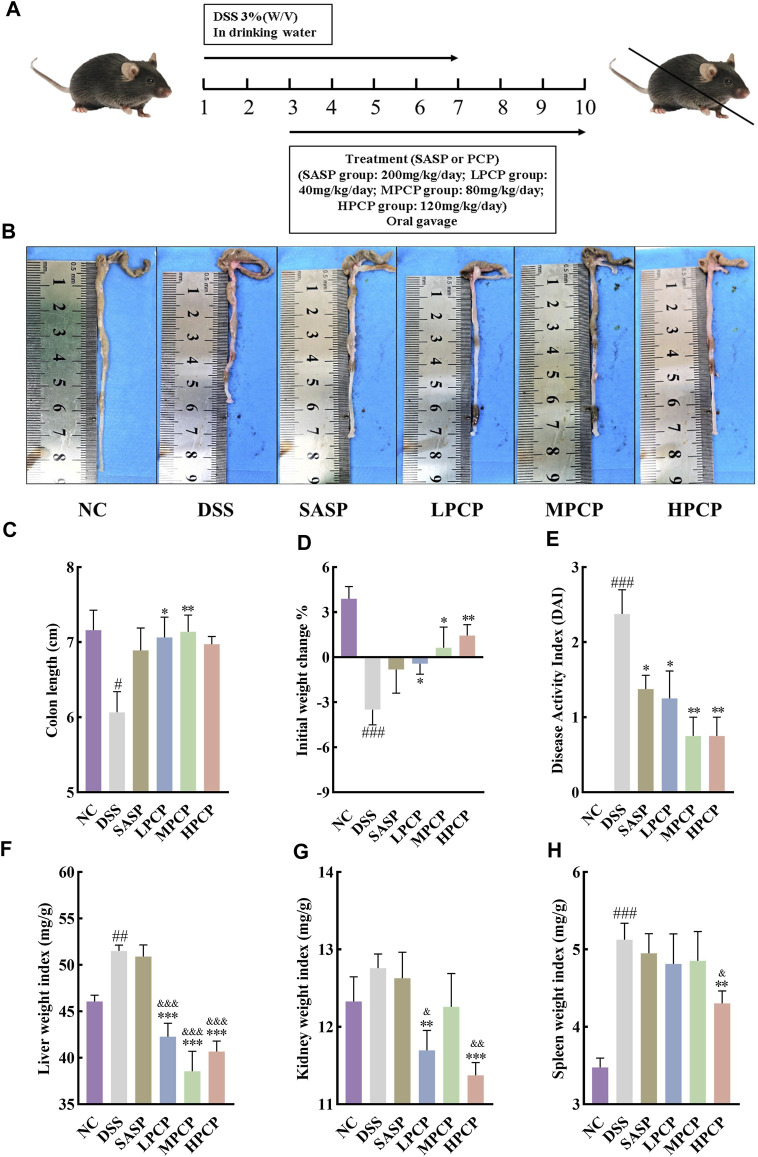
PCPs alleviate pathogenic phenotypic changes in DSS-induced UC mice (*n* = 8). **(A)** Schematic representation of animal experimental groups. **(B, C)**: Quantitative analysis of changes in colon lengths of mice in response to DSS. **(D)** Effect of DSS on the body weights of mice. **(E–H)**: Evaluation of the pathologic changes in mouse colon based on the DAI scores and organ indices of liver, kidneys, and spleen of mice among different groups. Data are presented as mean ± SEM. The experimental groups include NC (normal control mice), DSS (UC model mice), SASP (UC mice treated with 200 mg/kg SASP as a positive control), LPCP (UC mice treated with 40 mg/kg PCPs), MPCP (UC mice treated with 80 mg/kg PCPs), and HPCP (UC mice treated with 120 mg/kg PCPs). ^#^ *p <* 0.05, ^##^ *p <* 0.01, and ^###^ *p <* 0.001 vs. NC, ^*^ *p <* 0.05, ^**^ *p <* 0.01, and ^***^ *p <* 0.001 vs. DSS, ^&^ *p <* 0.05, ^&^ *p <* 0.01, and ^&^ *p <* 0.001 vs. SASP. UC: ulcerative colitis, DSS: dextran sodium sulfate, PCP: *Polygonatum cyrtonema* polysaccharide, DAI: disease activity index, SASP: sulfasalazine, LPCP: low-dose PCP, MPCP: medium-dose PCP, HPCP: high-dose PCP.

Female C57BL/6J mice (6 weeks old) were purchased from Zhejiang Vital River Laboratory Animal Technology Co., Ltd. (Jiaxing, China, Approval Number: SCXK (Zhe) 2019-0001). All animal experiments in this study were approved by the Animal Ethics Committee of Wenzhou Medical University and conducted in strict accordance with the standards outlined in the Guide for the Care and Use of Laboratory Animals. The mice were housed in a specific pathogen-free (SPF) animal facility and allowed to acclimatize for a week. Subsequently, the mice were randomly divided into six groups (*n* = 8, Figure 1A): Normal control (NC) group mice were given free access to distilled water and gavaged once daily with sterile water, and dextran sodium sulfate (DSS) group (model group) mice were provided free access to 3% DSS and gavaged once daily with sterile water. Sulfasalazine (SASP) group mice were given free access to 3% DSS and gavaged once daily with 200 mg/kg SASP. Low-dose PCP (LPCP) group mice were given free access to 3% DSS and gavaged once daily with 40 mg/kg PCPs, and medium-dose PCP (MPCP) group mice were provided free access to 3% DSS and gavaged once daily with 80 mg/kg PCPs. High-dose PCP group (HPCP) mice were given free access to 3% DSS and gavaged once daily with 120 mg/kg PCPs. The body weight, stool consistency, and rectal bleeding were evaluated daily during the experimental period. The mice were euthanized with pentobarbital sodium anesthesia after 7 days of drug treatment. Blood samples were collected from mice from different groups, sera separated, and samples were stored at −80°C. The liver, kidneys, and spleen from each mouse were excised, weighed, and used to calculate organ indices. The colon was excised from each mouse, photographed, and its length was measured. A portion of the colon was fixed in 4% paraformaldehyde for 24 h and embedded in paraffin for histopathologic analysis.

### 2.5 Pathologic scoring

The disease activity index (DAI) score was calculated as the sum of the scores for three symptoms of UC, namely, body weight loss, stool consistency, and rectal bleeding (Pan et al., 2022). Table 1 shows the DAI scoring criteria.

**TABLE 1:** DAI scoring scale.

DAI scores	Body weight loss (%)	Stool consistency	Bloody stools
0	0–1	Normal	No bleeding
1	1–5		
2	5–10	Soft stool	Slight bleeding
3	10–20		
4	≥20	Diarrhea	Gross bleeding

### 2.6 Hematoxylin and eosin (H&E) and Masson’s trichrome staining

The distal colon segment of approximately 1 cm was fixed in 4% paraformaldehyde for 24 h, embedded in paraffin, and sectioned into 4-μm thick slices. The sections were deparaffinized, stained with H&E and Masson’s trichrome stains, dehydrated, mounted on slides, and imaged under an upright fluorescence microscope.

### 2.7 Western blot assay

Total proteins were extracted from mouse tissues using a mammalian tissue protein extraction reagent. The total protein content of the tissue lysates was determined using a bicinchoninic acid protein assay kit. The extracted proteins were boiled in a sample buffer at 100°C for 10 min and separated using 10% sodium dodecyl sulfate–polyacrylamide gel electrophoresis. The separated proteins were then transferred onto polyvinylidene fluoride membranes. The membranes were blocked with 5% skim milk in TBST buffer at room temperature for 2 h. The membranes were probed with primary antibodies against ZO-1 (1:1000), occludin (1:1000), claudin (1:1000), α-SMA (1:4000), TGF-β1 (1:4000), and GAPDH (1:5000) overnight at 4°C followed by incubation with horseradish peroxidase-conjugated secondary antibodies (1:10000) at room temperature for 1 h. The enhanced chemiluminescence detection reagent was added to the membranes, and the blots were imaged using an Amersham Imager 680 ultra-sensitive chemiluminescence imaging system. The grayscale analysis of the images was performed using the ImageJ software.

### 2.8 Quantitative real-time polymerase chain reaction (qRT-PCR)

Total RNA from colon tissue was extracted using RNA isolater Total RNA Extraction Reagent and quantified using a Nanodrop microvolume spectrophotometer. Total RNA was then reverse transcribed into cDNA using the HiScript^®^ III All-in-one RT SuperMix Perfect for qPCR kit. qRT-PCR was performed on an ABI fluorescent quantitative PCR instrument using Taq Pro Universal SYBR qPCR Master Mix. The fold change in PCR analysis was determined using the 2^−ΔΔCT^ method. The primers used for qRT-PCR are listed in Table 2.

**TABLE 2:** qRT-PCR primer sequences.

Genes	Forward Primer (5′-3′)	Reverse Primer (5′-3′)
*IL-1β*	GCA​ACT​GTT​CCT​GAA​CTC​AAC​T	ATC​TTT​TGG​GGT​CCG​TCA​ACT
*IL-6*	TAG​TCC​TTC​CTA​CCC​CAA​TTT​CC	TTG​GTC​CTT​AGC​CAC​TCC​TTC
*TNF-α*	CCC​TCA​CAC​TCA​GAT​CAT​CTT​CT	GCT​ACG​ACG​TGG​GCT​ACA​G
*IL-10*	GAC​TTC​ACC​ATG​GAA​CCC​GT	GGA​GAC​TGC​CCA​TTC​TCG​AC
*GAPDH*	AGG​TCG​GTG​TGA​ACG​GAT​TTG	TGT​AGA​CCA​TGT​AGT​TGA​GGT​CA

### 2.9 Enzyme-linked immunosorbent assay (ELISA)

The relative serum concentrations of superoxide dismutase (SOD), glutathione (GSH), and MDA in mice were determined using ELISA kits according to the manufacturer’s instructions. The serum concentrations of cytokines were calculated based on the standard curves.

### 2.10 Macrogenomic analysis

Total microbial genomic DNA samples were extracted using the OMEGA Mag-Bind Soil DNA Kit (M5635-02) (Omega Bio-Tek, Norcross, GA, United States), following the manufacturer’s instructions, and stored at −20°C prior to further assessment. The quantity and quality of extracted DNAs were measured using a Qubit™ 4 Fluorometer, with WiFi: Q33238 (Qubit™ Assay Tubes: Q32856, Qubit™ 1X dsDNA HS Assay Kit: Q33231) (Invitrogen, United States) and agarose gel electrophoresis, respectively. The extracted microbial DNA was processed to construct metagenome shotgun sequencing libraries with insert sizes of 400 bp by using Illumina TruSeq Nano DNA LT Library Preparation Kit. Each library was sequenced by Illumina NovaSeq platform (Illumina, United States) with PE150 strategy at Personal Biotechnology Co., Ltd. (Shanghai, China).

### 2.11 Untargeted metabolomic analysis

Metabolite extraction from mouse feces and subsequent liquid chromatography–mass spectrometry (LC–MS) were performed by Personal Biotechnology Co., Ltd.

### 2.12 Assessment of the efficacy of PCP fractions (PCP-1 and PCP-2) and unfractionated PCPs

Female C56BL/6J mice (6 weeks old) were acclimatized for a week and divided into five groups (*n* = 6), namely, NC, DSS (model), PCP-1 (treated with PCP-1 at 80 mg/kg), PCP-2 (treated with PCP-2 at 80 mg/kg), and PCP (treated with PCPs at 80 mg/kg) groups. The mice in the DSS, PCP-1, PCP-2, and PCP groups were treated with 3% DSS in drinking water for 7 days to establish the UC model. The NC group mice received sterile water. The PCP-1, PCP-2, and PCP groups were force-fed the respective PCP fractions (80 mg/kg) from the third day for the next 7 days. The NC and DSS groups were force-fed with an equivalent amount of sterile water for 7 days.

### 2.13 Depletion of gut microbiota

Female C56BL/6J mice (4 weeks old) were acclimatized for 1 week and divided into three groups (*n* = 6), namely, DSS + PCP (treated with DSS and PCPs), DSS + ABX (treated with DSS and ABX), and DSS + ABX + PCP (treated with DSS, ABX, and PCP) groups. The mice in the DSS + ABX and DSS + ABX + PCP groups were allowed to drink water supplemented with ABX for 28 days (the ABX solution was changed every 2 days). The mice in the DSS group + PCP group were allowed to drink sterile water for 28 days. All three groups were then allowed to freely drink water containing 3% DSS for 7 days to induce UC. On the third day of DSS feeding (day 31), the mice in the DSS + PCP and DSS + ABX + PCP groups were orally gavaged with PCPs (80 mg/kg) for 7 days, whereas those in the DSS + ABX group were gavaged with an equal volume of sterile water for 7 days. The ABX solution was prepared by dissolving ampicillin (1.0 g/L), neomycin sulfate (1.0 g/L), metronidazole (1.0 g/L), and vancomycin (0.5 g/L) in sterile water.

### 2.14 Fecal microbiota transplantation (FMT)

Female C56BL/6J mice (6 weeks old) were divided into four groups (*n* = 6), namely, NC, DSS (model), FMT + NC (received fecal microbiota from the NC mice), and FMT + PCP (received fecal microbiota from the PCP-treated mice) groups. Fresh feces were collected from the donor mice in the NC and PCP-treated groups. The feces were then placed into sterile Eppendorf tubes and diluted with sterile saline solution (200 mg/2 mL). The mixture was vortexed for 5 min to create a homogeneous suspension, which was centrifuged at 600 g for 5 min to obtain a fecal suspension. This fecal suspension was orally administered to SPF UC mice (200 μL per mouse) to establish the gut microbiota. An equal volume of sterile saline solution was administered by oral gavage to the NC mice. The NC and DSS groups were administered physiologic saline, the FMT + NC group was given fecal suspension from donors in the NC group, and the FMT + PCP group was administered fecal suspension from donors in the PCP group.

### 2.15 16SrRNA gene sequence analysis

Mouse faecal samples were collected in a sterile environment and immediately frozen in liquid nitrogen at −80°C before storage. Total genomic DNA samples were extracted using the OMEGA Soil DNA Kit (M5636-02) (Omega Bio-Tek, M5636-02) (Omega Bio-Tek, Norcross, GA, United States) and stored at −20°C prior to further analysis. The quantity and quality of extracted DNA was measured using a NanoDrop NC2000 spectrophotometer (Thermo Fisher Scientific, Wallsburg). The V3-V4 region of the microbesl 16S rRNA gene was PCR amplified and sequenced on the Illumina NovaSeq platform using forward primer 338F (5′-ACT​CCT​ACG​GGA​GGC​AGC​A-3′) and reverse primer 806R (5′-GGACTACHVGGTWTCTAAT-3′) (Shanghai Personal Biotechnology Co., Ltd.).

### 2.16 Data analysis

Data were statistically analyzed using the GraphPad Prism 8.0 software. All data were expressed as mean ± SEM. One-way analysis of variance (ANOVA) and independent sample *t*-test were performed to compare different groups. A *p*-value of <0.05 was considered statistically significant.

## 3 Results

### 3.1 PCPs alleviate pathogenic phenotypic changes in DSS-induced UC mice

DSS stimulation notably decreased colon length and body weight and increased liver and spleen weight indices in the model group compared with the NC group (Figures 1B–D, F, H, *p <* 0.05). However, no significant change was noted in the kidney weight index (Figure 1G). The DAI scores, calculated by assessing fecal characteristics, occult blood, body weight, and colon length, showed a significant increase in DSS-treated mice compared with NC mice (Figure 1E, *p <* 0.001). However, SASP treatment only significantly reversed the increase in the DAI scores in UC mice (Figure 1E, *p <* 0.05). PCPs effectively restored colon length, mitigated body weight loss, lowered the DAI scores, and decreased weight indices of the liver, kidneys, and spleen in UC mice (Figures 1B–H, *p <* 0.05). We compared different characteristics of UC in the three PCP treatment groups and found that the reversal in decreased body weight and increased kidney and spleen indices was superior in the HPCP group, whereas reversal in decreased colon length and increased DAI score and liver weight index was better in the MPCP group.

### 3.2 PCPs attenuate colon tissue damage in UC mice

**FIGURE 2:**
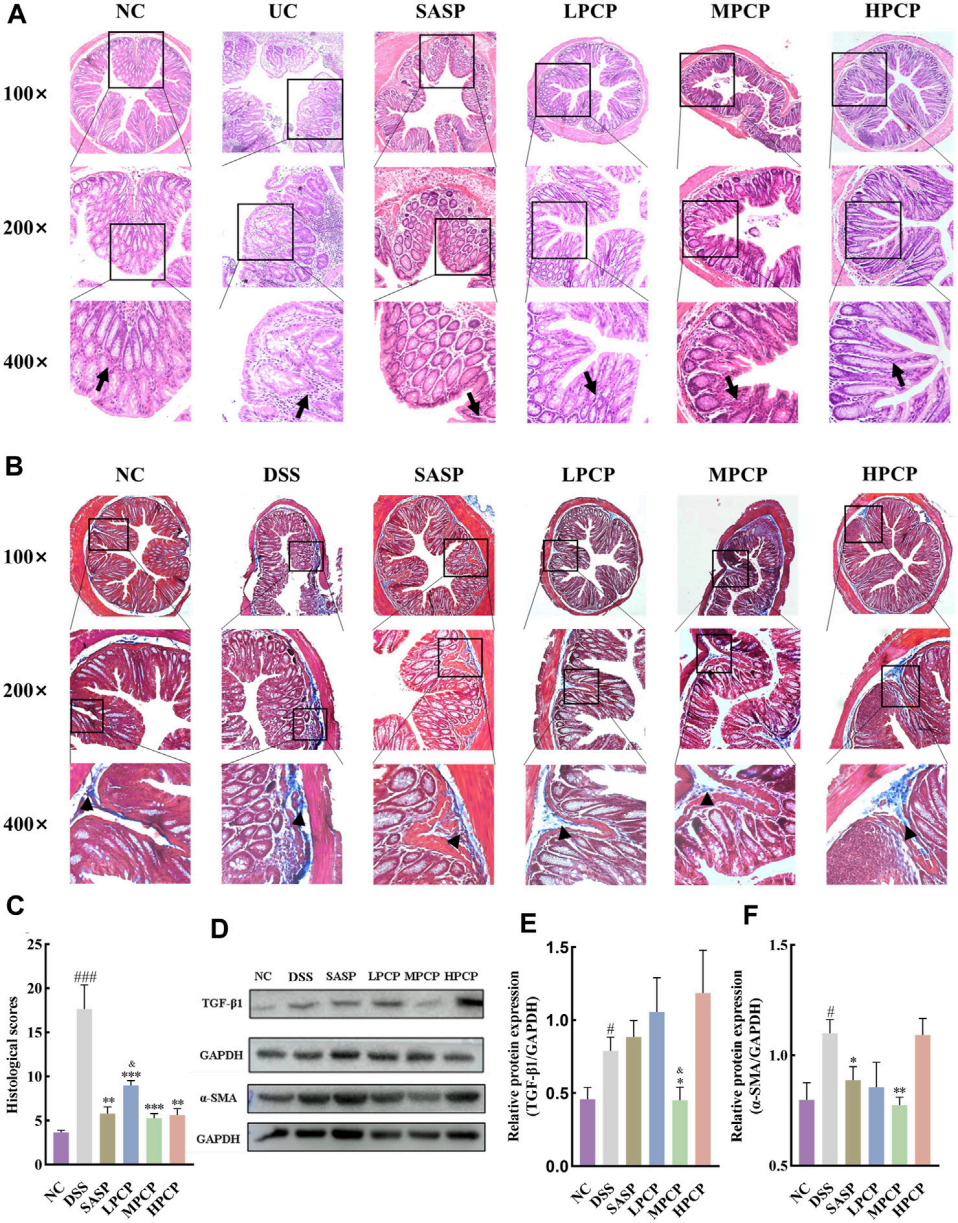
PCPs alleviate colon tissue damage in DSS-induced UC mice (*n* = 4/5). **(A, B)**: H&E and Masson’s trichrome staining micrographs of the paraffin sections of colon tissues from mice at 100×, ×200, and ×400 magnifications show inflammatory cell infiltration (➔) and collagen deposition (▲) after DSS treatment. **(C)**: Collagen volume fractions in colon tissues of mice from each group determined using Masson’s trichrome staining. **(D–F)**: Protein levels of TGF-β1 and α-SMA in colon tissues of mice from each group, with GAPDH as the loading control. Data are presented as mean ± SEM. ^#^ *p <* 0.05, ^##^ *p <* 0.01, and ^###^ *p <* 0.001 vs. NC, ^*^ *p <* 0.05, ^**^ *p <* 0.01, and ^***^ *p <* 0.001 vs. DSS, ^&^ *p <* 0.05 vs. SASP. UC: ulcerative colitis, DSS: dextran sodium sulfate, PCP: *Polygonatum cyrtonema* polysaccharide.

H&E staining images showed that the cross-section of the colon of mice lost its elliptical shape in the DSS group compared with the NC group, indicating severe damage to the colon structure. The images also indicated a significant reduction in the number of goblet cells, the disappearance of crypts, the presence of high mucoid content in the colonic lumen, pronounced infiltration of inflammatory cells, severe tissue edema, the presence of focal ulcers, and disruption of the tissue structure (Figure 2A). Masson’s trichrome staining images revealed an increase in the fibrotic area in the colon of mice in the DSS group compared with the NC group (Figures 2B,C, *p <* 0.001). Western blot results showed that the levels of TGF-β1 and α-SMA in the colon tissues of mice were increased after DSS administration in the model group compared with the NC group (Figures 2D–F, *p <* 0.05). SASP treatment ameliorated colon tissue damage (Figure 2A), reduced fibrotic area (Figures 2B,C, *p <* 0.01), and decreased α-SMA levels (Figures 2D,F, *p <* 0.05) in UC mice, however, TGF-β1 levels remained unchanged (Figures 2D,E). Conversely, effective amelioration of colon tissue damage (Figure 2A) and a significant reduction in the fibrotic area (Figures 2B,C) were observed after PCP treatment. However, the levels of TGF-β1 and α-SMA were significantly decreased after MPCP treatment (Figures 2D–F, *p <* 0.05).

### 3.3 PCPs ameliorate inflammation, oxidative stress, and intestinal mucosal injury in UC mice *in vivo*

**FIGURE 3:**
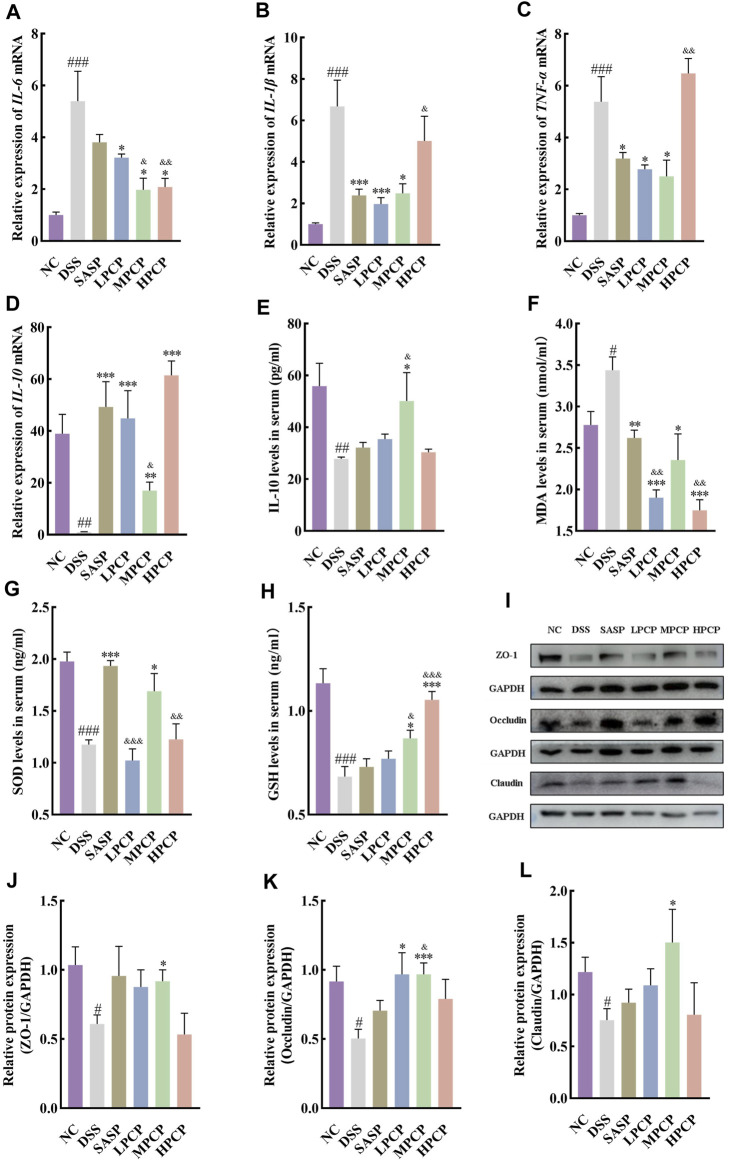
PCPs ameliorate inflammation, oxidative stress, and intestinal mucosal injury in UC mice (*n* = 4/5). **(A–D)**: mRNA expression of *IL-6*, *IL-1β*, *TNF-α*, and *IL-10* in colon tissues of mice from each group with *GAPDH* as the housekeeping gene. **(E–H)**: Serum levels of IL-10, MDA, SOD, and GSH in mice from each group. **(I–L)**: Protein levels of ZO-1, occludin, and claudin in colon tissues of mice from each group, with GAPDH as the loading control. Data are presented as mean ± SEM. ^#^ *p <* 0.05, ^##^ *p <* 0.01, and ^###^ *p <* 0.001 vs. NC, ^*^ *p <* 0.05, ^**^ *p <* 0.01, and ^***^ *p <* 0.001 vs. DSS, ^&^ *p <* 0.05, ^&^ *p <* 0.01, and ^&^ *p <* 0.001 vs. SASP. UC: ulcerative colitis, DSS: dextran sodium sulfate, PCP: *Polygonatum cyrtonema* polysaccharide.

DSS-treated mice showed a significant increase in the expression of *IL-6*, *IL-1β*, and *TNF-α* mRNA and a decrease in that of *IL-10* mRNA in mouse colon tissues compared with the NC group (Figures 3A–D, *p <* 0.001). Furthermore, the serum levels of IL-10 decreased in DSS-treated mice (Figure 3E, *p <* 0.01). SASP treatment significantly reduced the expression of *IL-1β* and *TNF-α* mRNA in mouse colon tissues (Figures 3B,C, *p <* 0.05). However, the treatment did not alter the expression of *IL-6* mRNA (Figure 3A, *p* > 0.05). Moreover, SASP treatment significantly increased the expression of *IL-10* mRNA in mouse colon tissues (Figure 3D, *p <* 0.001). However, serum IL-10 concentrations were unchanged (Figure 3E). The expression of *IL-6* and *TNF-α* mRNA was significantly reduced in colon tissues of UC mice from the MPCP group compared with those from other two PCPs treatment groups. In contrast, the expression of *IL-10* mRNA in colon tissues and serum IL-10 levels significantly increased in the MPCP group (Figures 3A–E, *p <* 0.05). LPCP and HPCP partially counteracted the changes in inflammatory factors in UC mice.

Furthermore, serum levels of MDA (Figure 3F, *p <* 0.05) were significantly increased and those of SOD (Figure 3G, *p <* 0.001) and GSH (Figure 3H, *p <* 0.001) were decreased in the DSS-treated group (model group) compared with the NC group. SASP treatment significantly decreased the serum levels of MDA and markedly increased SOD levels (Figures 3F,G, *p <* 0.05) in mice. However, the treatment had no significant impact on GSH concentrations (Figure 3H). Oxidative stress was decreased in the LPCP, MPCP, and HCP groups. HPCP exhibited a more pronounced effect on restoring GSH levels, while MPCP showed a superior effect on restoring MDA and SOD levels (Figures 3F–H, *p <* 0.05). Western blot results revealed that the expression levels of intestinal tight junction proteins ZO-1, occludin, and claudin were significantly decreased in the colon tissues of DSS-treated mice (Figures 3I–L, *p <* 0.05). SASP treatment did not affect the levels of ZO-1, occludin, and claudin in mouse colon tissues. However, MPCP treatment significantly increased the levels of these tight junction proteins in the colon tissue of UC mice (Figures 3I–L, *p <* 0.05).

Taken together, the therapeutic effect of MPCP was better than that of SASP and other PCP doses (LPCP and HPCP). Therefore, an 80 mg/kg dose of PCP was used in subsequent experiments.

### 3.4 PCP regulates the structure and function of the gut microbiota in DSS-induced UC mice

Mouse fecal metagenomic analysis was conducted to evaluate the effect of DSS, SASP, and PCP treatments on the composition and function of the gut microbiota. Alpha diversity analysis revealed that mice in the DSS group exhibited a significant reduction in both Simpson and Shannon indices compared with those in the NC group (Supplementary Figures S1B, C, *p <* 0.05), whereas the Chao1, observed-species, and ACE indices were not significantly different between the two groups (Supplementary Figures S1A, D, E). SASP treatment resulted in a significant increase in the Chao1, observed-species, and ACE indices (*p <* 0.05), whereas the Shannon and Simpson indices showed no significant changes. Notably, the Chao1, Simpson, Shannon, observed-species, and ACE indices were restored after PCP treatment (Supplementary Figures S1A–E, *p <* 0.05), indicating an increase in the abundance of intestinal microbiota. The principal coordinate analysis (PCoA) of β-diversity based on unweighted UniFrac distance revealed significant differences in gut microbiota composition among the NC, DSS, SASP, and MPCP groups (Supplementary Figure S1F).

The distribution of dominant phyla, genera, and species among the four treatment groups was illustrated using bar plots and heatmaps (Supplementary Figures S1G–I). The dominant phyla included Bacteroidota (53.1%), Firmicutes (34.7%), Verrucomicrobiota (5.41%), Chordata (0.86%), and Desulfobacterota I (1.93%) in the NC group. The DSS group showed a significant reduction in the abundance of Verrucomicrobiota (0.004%) and Chordata (0.015%) compared with the NC group (*p <* 0.01). The abundance of Desulfobacterota I (0.18%) was significantly increased in the SASP group. However, no significant effects were observed on the abundance of Verrucomicrobiota (0.039%) and Chordata (1.32%). The abundance of Verrucomicrobiota (4.98%) and Firmicutes B (0.039%) was significantly increased, and no significant changes were observed in the abundance of *Chordata* (0.135%) and Desulfobacterota I (0.017%) in the MPCP group (Supplementary Figure S1J).

*Muribaculum* (8.14%), *UBA3263* (2.48%), *CAG-873* (4.43%), *Alistipes* (1.86%), *CAG-485* (12.79%), *Duncaniella* (10.77%), and *Akkermansia* (5.41%) were the dominant genera in the NC group. Relative to the NC group, the DSS group showed a significant decrease in the abundance of *Muribaculum* (0.246%), *Duncaniella* (2.95%), and *Akkermansia* (0.004%, *p <* 0.05), whereas a significant increase was observed in the abundance of *UBA3263* (16.67%) and *CAG-873* (12.06%) (*p <* 0.05). The abundance of *Muribaculum* (14.26%) was significantly increased in the SASP group (*p <* 0.05), whereas the abundance of *Duncaniella* (2.06%), *Akkermansia* (0.04%), *UBA3263* (6.84%), and *CAG-873* (5.72%) demonstrated no significant alterations. MPCP treatment significantly elevated the abundance of *Akkermansia* (4.98%) (*p <* 0.01) and diminished the abundances of *UBA3263* (1.32%) and *CAG-873* (3.49%) (*p <* 0.01), whereas no significant changes were observed in the abundance of *Muribaculum* (1.37%) and *Duncaniella* (3.46%, Supplementary Figure S1K).

The dominant species in the NC group included *UBA3263* sp001689615 (2.48%), *Bacteroides caecimuris* (0.037%), *CAG-873* sp910587235 (0.348%), *A. muciniphila* A (4.49%), *Alistipes* sp910577475 (0.009%), and *UBA7173* sp013316535 (1.08%). Relative to the NC group, the DSS group showed a significant decrease in the abundance of *A. muciniphila* A (0.0008%, *p <* 0.01) and a significant increase in the abundance of *UBA3263* sp001689615 (16.67%), *CAG-873* sp910587235 (8.59%), and *UBA7173* sp013316535 (5.76%, *p <* 0.05). SASP treatment significantly decreased the abundance of *CAG-873* sp910587235 (1.08%) (*p <* 0.05), whereas the abundance of *A. muciniphila* A (0.0322%), *UBA3263* sp001689615 (6.84%), and *UBA7173* sp013316535 (2.18%) was not significantly altered. MPCP treatment significantly enhanced the abundance of *A. muciniphila* A (4.15%) (*p <* 0.001) and diminished that of *UBA3263* sp001689615 (1.32%), *CAG-873* sp910587235 (0.19%), and *UBA7173* sp013316535 (2.17%) (Supplementary Figure S1L, *p <* 0.05).

Species functional beta diversity analysis revealed differences in species function among the four treatment groups across various databases (Supplementary Figure S2). The distribution and differences in species function among the four treatment groups in various databases are illustrated in bar plots and heatmaps (Supplementary Figures S3A–D). The KEGG database analysis revealed that the abundance of species on transposase (K07484) was significantly increased in the MPCP group compared with the NC group (*p <* 0.05), whereas significant differences were not observed among the DSS, SASP, and MPCP groups (Supplementary Figure S3E). The eggNOG database analysis revealed that species abundance on reverse transcriptase (RNA-dependent DNA polymerase, 3BK61@33208) was significantly decreased in the DSS group compared with the NC group (*p <* 0.01). The SASP and MPCP treatments led to an increase in species abundance in this pathway. However, the differences were not statistically significant (Supplementary Figure S3F). The GO database analysis revealed that the species abundance in the “viral process, biological-process” category (GO: 0016032) significantly decreased in the DSS group compared with the NC group (*p <* 0.05). After SASP treatment, an increase was noted in the species abundance in this pathway. However, the differences were not statistically significant (Supplementary Figure S3G). Furthermore, the SwissProt database analysis revealed that species abundance in “Putative transposase (P55729.1)” and “Nitrite reductase, large subunit, NAD(P)H-binding (P50360.2)” increased in the DSS group compared with the NC group (*p <* 0.05). After SASP treatment, a significant reduction was found in the species abundance in “Uncharacterized protein in vnfD 5′region (P24423.1)” and “Nitrite reductase, large subunit, NAD(P)H-binding” (*p <* 0.05). In contrast, the species abundance in “Nitrite reductase, large subunit, NAD(P)H-binding” was significantly decreased after MPCP treatment (*p <* 0.01) without causing significant changes in “Putative transposase” and “Uncharacterized protein in vnfD 5’region” (Supplementary Figure S3H).

### 3.5 PCPs alter the fecal metabolome of DSS-induced UC mice

LC–MS analysis revealed 1,102 metabolites in the feces of mice from the NC, DSS, SASP, and MPCP groups. Multivariate statistical analysis, including principal fraction analysis (PCA), partial least squares-discriminant analysis (PLS–DA), and orthogonal PLS–DA (OPLS–DA), revealed notable separation among various treatments, indicating significant differences in colonic metabolism in response to DSS, SASP, and PCP treatments (Supplementary Figure S4; Supplementary Figures S5A,B).

Compared with the NC group, the DSS-treated group showed 404 differentially abundant metabolites (328 upregulated and 76 downregulated, Table 3). Furthermore, “N5-Methyl-L-glutamine,” “Biochain A,” “Porphobilinogen,” “Imperatorin,” and “Gamabufogenin” were downregulated in the NC group but upregulated in the DSS group (Supplementary Figures S5C (I)). KEGG pathway analysis indicated that differentially abundant metabolites were primarily enriched in pathways related to “Steroid hormone biosynthesis,” “Prostate cancer,” “Cholesterol metabolism,” “Linoleic acid metabolism,” and “Arachidonic acid metabolism” (Supplementary Figures S5D (I)). The comparison of DSS and SASP groups revealed that SASP treatment induced 115 differentially abundant metabolites, including 51 upregulated and 64 downregulated metabolites (Table 3). “1-Hexadecylthio-2-hexadecanoylamino-1, 2-dideoxy-sn-glycerol-3-phosphocholine” and “D-Ornithine hydrochloride” were downregulated in the DSS group but upregulated in the SASP group. In contrast, “5′-Dehydroadenosine,” “4, 5-Dihydroorotic acid,” and “Andrographolide” were upregulated in the DSS group but downregulated in the SASP group (Supplementary Figure S5C (II)). These metabolites were primarily enriched in pathways related to “Caffeine metabolism,” “Vascular smooth muscle contraction,” “Arachidonic acid metabolism,” “Phenylalanine metabolism,” and “Serotonergic synapse” (Supplementary Figure S5D (II)). The comparison of DSS and MPCP groups revealed that PCP treatment induced 207 differentially abundant metabolites, including 63 upregulated and 144 downregulated metabolites (Table 3). “Gamabufogenin,” “Pentaporphyrin I,” “Eriodictyol,” “Hydroxykynurenine,” and “Antheraxanthin” were upregulated in the DSS group but downregulated in the MPCP group (Supplementary Figure S5C (III)). These metabolites were primarily enriched in pathways associated with “Nicotine addiction,” “Retrograde endocannabinoid signaling,” “Nicotinate and nicotinamide metabolism,” “Prostate cancer,” and “Caffeine metabolism” (Supplementary Figure S5D (III)).

**TABLE 3:** Statistics of differential metabolites.

Comparison	Up	Down	Total
NC vs. DSS	328	76	404
DSS vs. SASP	51	64	115
DSS vs. MPCP	63	144	207

### 3.6 Correlation between fecal microbiota and metabolites in DSS-induced UC mice treated with PCPs

Mantel tests were performed to investigate the potential correlation between fecal microbiota species and metabolites. The results demonstrated a significant correlation between the genera and species present in fecal microbiota and metabolites (*p <* 0.05). A significant positive correlation was observed between *Duncaniella* and “Docosahexaenoic acid” at the genus level (*p <* 0.05). Furthermore, *Akkermansia* showed a significant positive correlation with “Gamabufogenin,” “Antheraxanthin,” “Porphobilinogen,” “Pentaporphyrin I,” “Biochanin A,” and “Eriodictyol” (*p <* 0.05). *Alistipes A* showed a significant positive correlation with “Gamabufogenin,” “Antheraxanthin,” “Porphobilinogen,” “N5-Methyl-L-glutamine,” “Pentaporphyrin I,” “5-Dehydroadenosine,” “Biochanin A,” “Bovinic acid,” “Eriodictyol,” “Imperatorin,” and “EPA (d5)” (*p <* 0.05) but a significant negative correlation with “L-Methionine” and “L-3-Hydroxykynurenine” (*p <* 0.05). *UBA3263* had a significant positive correlation with “Catechol” (*p <* 0.01) and a significant negative correlation with “N5-Methyl-L-glutamine” (*p <* 0.05). *CAG-873* was negatively correlated with “Gamabufogenin,” “Antheraxanthin,” “N5-Methyl-L-glutamine,” “Pentaporphyrin I,” “5-Dehydroadenosine,” “Apigenin,” “Eriodictyol,” and “Imperatorin” (*p <* 0.05). However, it was positively correlated with “Docosahexaenoic acid” and “1-Hexadecylthio-2-hexadecanoylamino-1, 2-dideoxy-sn-glycero-3-phosphocholine” (*p <* 0.05, Supplementary Figure S6A).

*UBA3263* sp001689615 showed a significant positive correlation with “Catechol” (*p <* 0.01) and a significant negative correlation with “N5-Methyl-L-glutamine” (*p <* 0.05) at the species level. *Bacteroides caecimuris* showed a significant negative correlation with “D-Ornithine hydrochloride” and “1-Hexadecylthio-2-hexadecanoylamino-1, 2-dideoxy-sn-glycero-3-phosphocholine” (*p <* 0.05). Furthermore, *CAG-873* sp910587235 demonstrated significant negative correlations with “Gamabufogenin,” “Antheraxanthin,” “Porphobilinogen,” “Andrographolide,” “N5-Methyl-L-glutamine,” “5-Dehydroadenosine,” “Biochanin A,” “Eriodictyol,” and “Imperatorin” (*p <* 0.05). It also showed significant positive correlations with “1-Hexadecylthio-2-hexadecanoylamino-1, 2-dideoxy-sn-glycero-3-phosphocholine,” “Docosahexaenoic acid,” and “L-3-Hydroxykynurenine” (*p <* 0.05). *A. muciniphila* A was positively correlated with “Gamabufogenin,” “Antheraxanthin,” “Porphobilinogen,” “Biochanin A,” and “Eriodictyol” (*p <* 0.05). *Alistipes* sp910577475 was negatively correlated with “Gamabufogenin,” “Porphobilinogen,” “Phytosphingosine,” “N5-Methyl-L-glutamine,” “1-Hexadecylthio-2-hexadecanoylamino-1, 2-dideoxy-sn-glycero-3-phosphocholine,” “Biochanin A,” “Bovinic acid,” “Imperatorin,” and “EPA (d5)" (*p <* 0.05) and positively correlated with “Catechol” (*p <* 0.05, Supplementary Figure S6B).

### 3.7 PCPs demonstrate superior therapeutic efficacy compared to PCP-1 and PCP-2

PCPs were fractionated using ion exchange chromatography to identify their specific fractions with therapeutic properties. Two molecular weight fractions, PCP-1 (19.9 kDa) and PCP-2 (71.6 and 4.2 kDa), were obtained after fractionation. Supplementary Figure S7A shows the monosaccharide composition analysis of the two PCP fractions. PCP-1 was composed of mannose, galacturonic acid, galactose, and xylose with the molar ratios of 0.02:0.01:1.00:0.05, and PCP-2 consisted of rhamnose, galacturonic acid, galactose, and xylose with the molar ratios of 0.19:0.43:1.00:0.24. The results of high-performance GPC indicated that the molecular weight of PCP-1 was 19.9 kDa, whereas PCP-2 had two molecular weight fractions of 71.6 and 4.2 kDa (Supplementary Figure S7B). The incomplete separation of polysaccharides in PCP-2 can be attributed to the use of ion exchange column chromatography as the purification method. The FT-IR analysis showed similar absorption peaks of PCP-1 and PCP-2 (Supplementary Figure S7C). The absorption peaks at 3312 cm^−1^ and 3340 cm^−1^ were attributed to the stretching vibrations of OH groups, and those at 2923 cm^−1^ and 2932 cm^−1^ corresponded to the stretching vibrations of CH groups. Peaks at 1600 cm^-1^ and 1621 cm^−1^ were associated with the stretching vibrations of C=O groups, and peaks at 1371 cm^−1^ and 1415 cm^−1^ were related to the stretching vibrations of C-H groups. The peaks at 1045 cm^−1^ and 1053 cm^−1^ were due to the stretching vibrations of C-O-C glycosidic bonds, and those at 890 cm^−1^ and 892 cm^−1^ were attributed to the stretching vibrations of the furanose ring of β-glycosidic bonds. The absorption peak at 770 cm^−1^ was associated with the stretching vibrations of furan rings (Gao et al., 2019; Xie et al., 2020; He et al., 2021). These results were consistent with the monosaccharide composition analysis depicted in Supplementary Figure S7A. Both PCP-1 and PCP-2 displayed no significant absorption peaks in the UV spectra between 260 and 280 nm (Supplementary Figure S7D), indicating the absence of proteins in these PCPs and ruling out the occurrence of glycoproteins in these fractions. The surface structure of polysaccharides is crucial to their biological functions. SEM images revealed distinct morphologic structures of the two purified PCP fractions (Supplementary Figure S7E). PCP-1 showed an irregular block-like surface with a smooth texture and some irregularly distributed large voids, suggesting a highly branched, entangled, and porous structure. In contrast, PCP-2 appeared as a sheet-like structure with an overall smooth surface and some minor wrinkles, apparently due to the lower branching degree of PCP-2 (Zhao et al., 2022).

**FIGURE 4:**
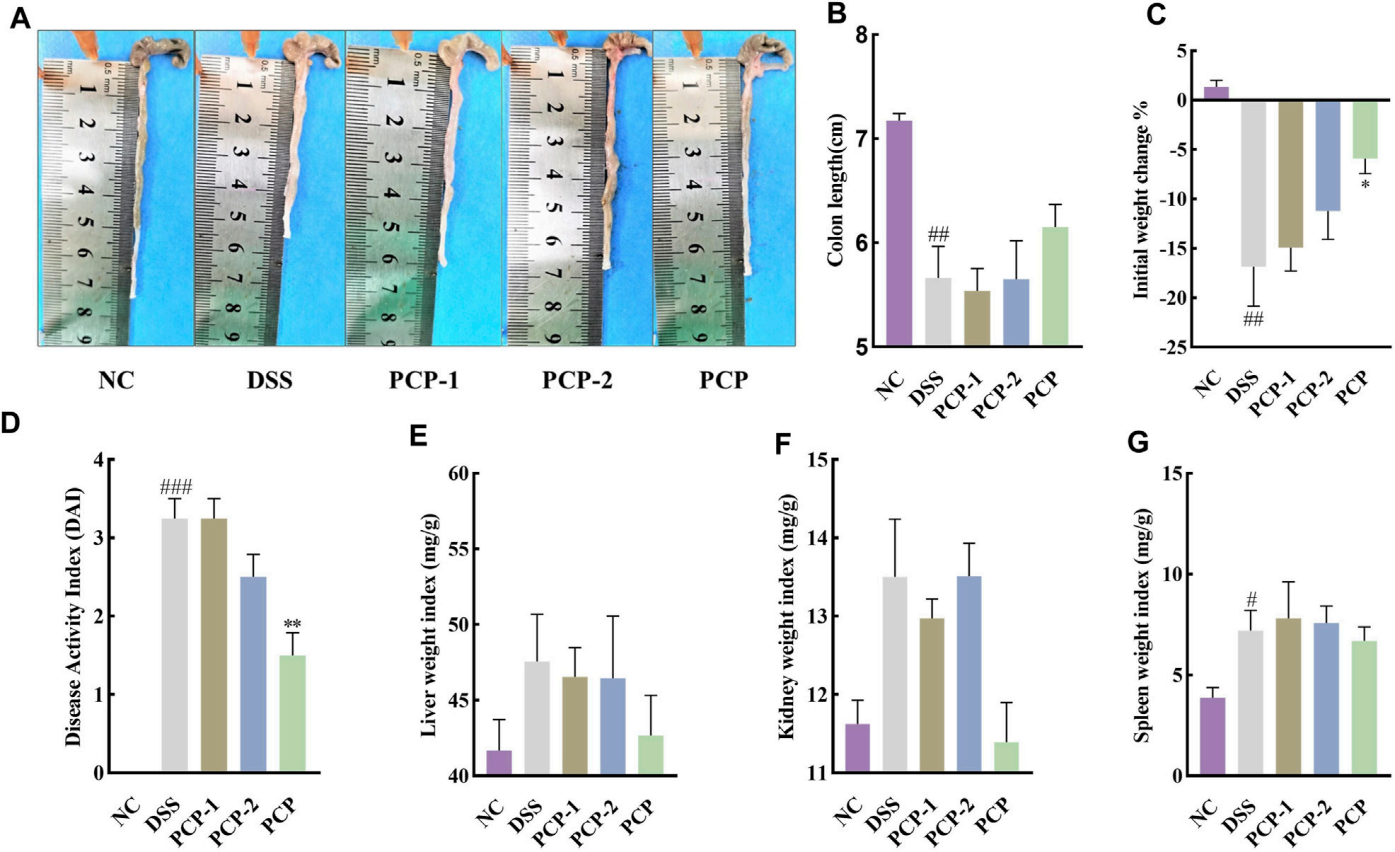
Unfractionated PCPs show superior therapeutic effects than fractionated fractions PCP-1 and PCP-2 in alleviating pathogenic phenotypic characteristics of DSS-induced UC mice (*n* = 4). **(A, B)**: Quantitative analysis of changes in colon lengths of mice in different groups. **(C)**: Quantitative analysis of body weight of mice in different groups. **(D)**: Evaluation of pathologic changes in mouse colon using the DAI scores. **(E–G)**: Organ indices of liver, kidney, and spleen of mice from different groups. Data are presented as mean ± SEM. ^#^ *p <* 0.05, ^##^ *p <* 0.01, and ^###^ *p <* 0.001 vs. NC, ^*^ *p <* 0.05 and ^**^ *p <* 0.01 vs. DSS. UC: ulcerative colitis, DSS: dextran sodium sulfate, PCP: *Polygonatum cyrtonema* polysaccharide.

**FIGURE 5:**
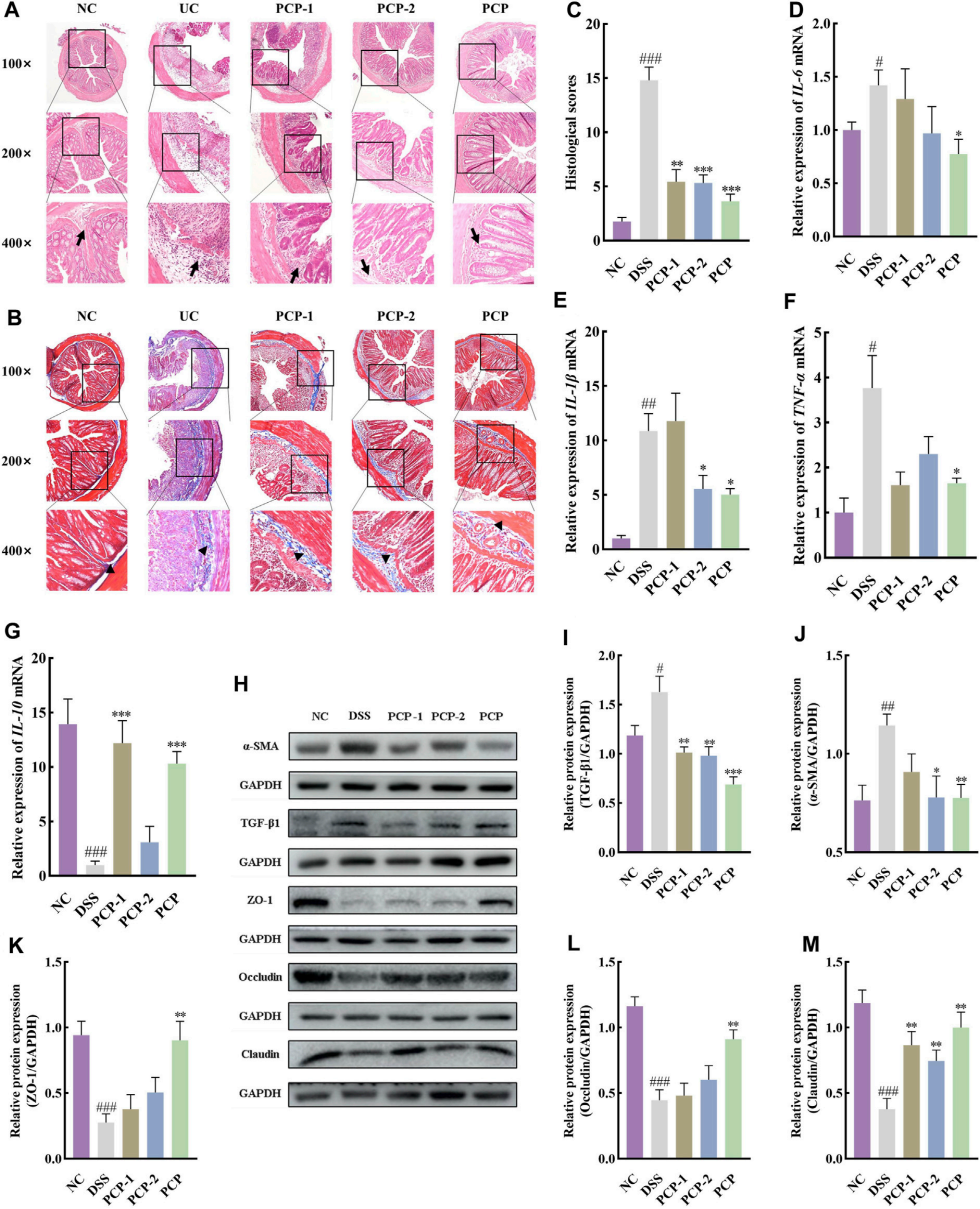
Unfractionated PCPs show superior therapeutic effects in alleviating colon tissue lesions, inflammatory imbalance, and mucosal injury in DSS-induced UC mice (*n* = 4). **(A, B)**: Microscopic images of H&E and Masson’s trichrome staining of colon tissue sections at 100×, ×200, and ×400 magnifications show inflammatory cell infiltration (➔) and collagen deposition (▲). **(C)**: Quantification of collagen volume fraction in colon tissues using Masson’s trichrome staining. **(D–G)**: Quantification of mRNA expression of *IL-6*, *IL-1β*, *TNF-α*, and *IL-10* in colon tissues of mice from each group (*GAPDH* is the housekeeping gene). **(H–M)**: Protein levels of α-SMA, TGF-β1, ZO-1, occludin, and claudin in colon tissues of mice from different groups, with GAPDH as the loading control. Data are presented as mean ± SEM. ^#^ *p <* 0.05, ^##^ *p <* 0.01, and ^###^ *p <* 0.001 vs. NC, ^*^ *p <* 0.05, ^**^ *p <* 0.01, and ^***^ *p <* 0.001 vs. DSS. UC: ulcerative colitis, DSS: dextran sodium sulfate, PCP: *Polygonatum cyrtonema* polysaccharide.

Unfractionated PCPs were superior to PCP-1 and PCP-2 in ameliorating the symptoms of DSS-induced UC in mice. PCPs had better therapeutic effects than their fractions in terms of increasing colon length, mitigating body weight loss, lowering the DAI scores, diminishing the weight indices of the liver and spleen (Figure 4), mitigating colonic damage (Figure 5A), and decreasing the concentration of fibrosis-associated proteins (α-SMA and TGF-β1) (Figures 5B,C, H–J). Similarly, PCPs were superior to PCP-1 and PCP-2 in regulating the mRNA expression of pro-inflammatory (*IL-6*, *IL-1β*, and *TNF-α*) and anti-inflammatory (*IL-10*) cytokines in the colon tissues (Figures 5D–G) and enhancing the expression of intestinal tight junction proteins (ZO-1, occludin, and claudin) (Figures 5H, K–M).

### 3.8 ABX treatment attenuates the therapeutic effect of PCPs in DSS-induced UC mice

**FIGURE 6:**
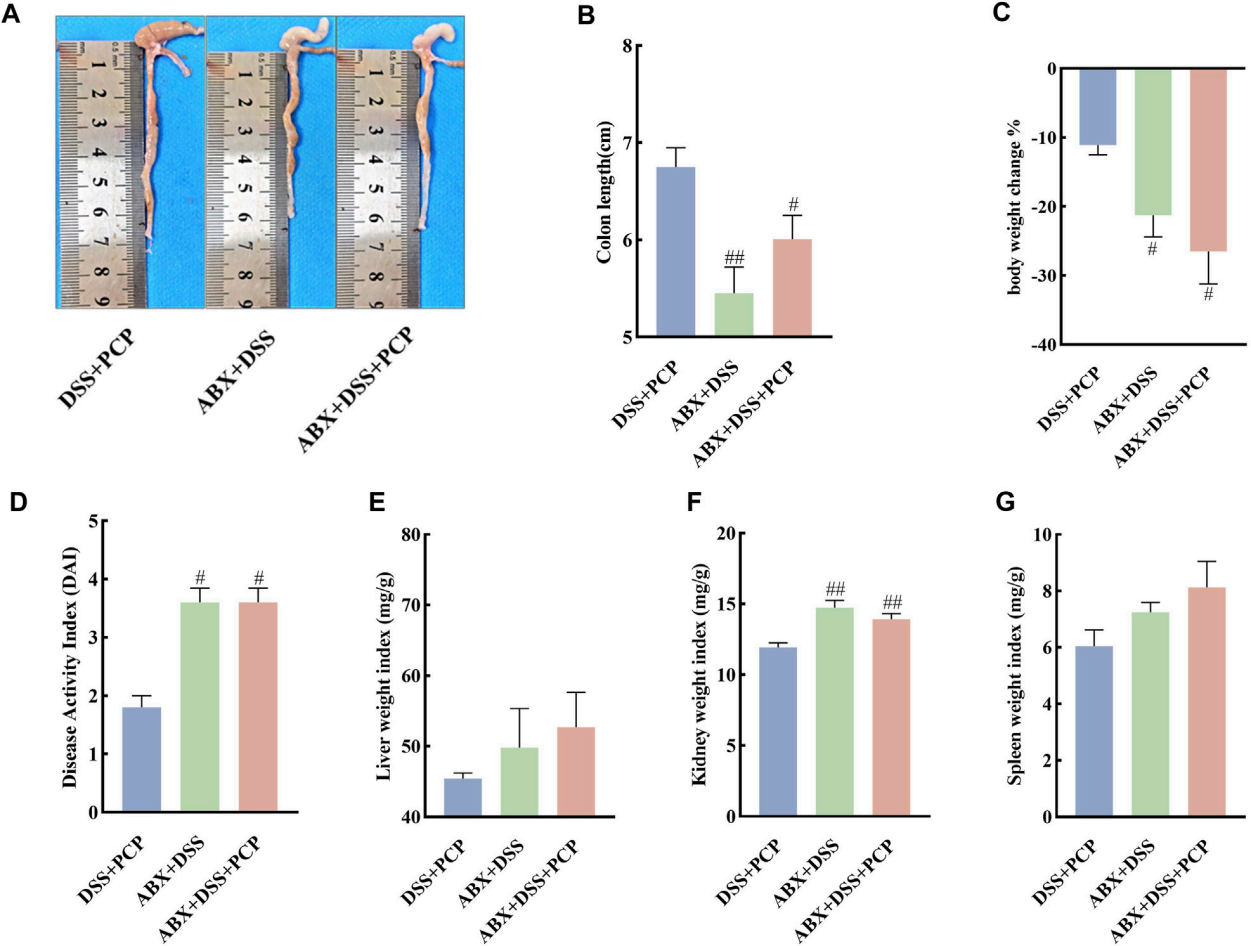
ABX treatment diminishes the therapeutic effects of PCPs on pathogenic phenotypic features of UC mice (*n* = 5). **(A, B)**: Quantitative analysis of colon lengths of mice in each group. **(C)**: Quantitative analysis of changes in body weights of mice in each group. **(D)**: Assessment of pathologic changes in mouse colon using the DAI scores of each group. **(E–G)**: Organ indices of liver, kidney, and spleen from mice in each group. Data are presented as mean ± SEM. ^#^ *p <* 0.05 and ^##^ *p <* 0.01 vs. DSS + PCP. UC: ulcerative colitis, DSS: dextran sodium sulfate, PCP: *Polygonatum cyrtonema* polysaccharide, ABX: antibiotics.

**FIGURE 7:**
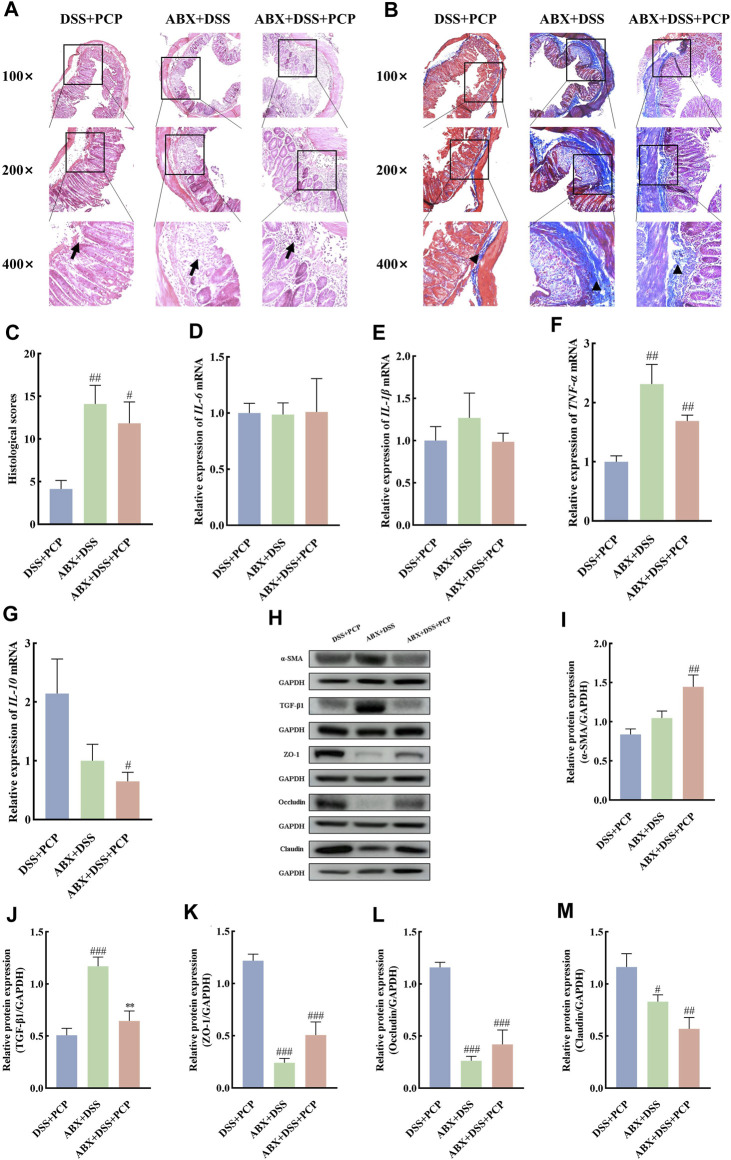
ABX treatment reduces the therapeutic effects of PCPs on colon tissue lesions, inflammatory imbalance, and mucosal injury in UC mice (*n* = 4/5). **(A, B)**: H&E staining and Masson’s trichrome staining images of colon tissue sections at 100×, ×200, and ×400 magnifications show inflammatory cell infiltration (➔) and collagen deposition (▲). **(C)**: Quantification of collagen volume fraction in mouse colon tissues using Masson’s trichrome staining. **(D–G)**: *IL-6*, *IL-1β*, *TNF-α*, and *IL-10* mRNA expression in colon tissues of mice, with *GAPDH* as the housekeeping gene. **(H–M)**: Protein levels of α-SMA, TGF-β1, ZO-1, occludin, and claudin in colon tissues of mice, with GAPDH as the loading control. Data are presented as mean ± SEM. DSS + PCP: DSS-exposed mice treated with PCPs, ABX + DSS, mice treated with ABX and DSS, ABX + DSS + PCP: mice treated with ABX, DSS, and PCPs. ^#^ *p <* 0.05, ^##^ *p <* 0.01, and ^###^ *p <* 0.001 vs. DSS + PCP, ^**^ *p <* 0.05 vs. ABX + DSS. UC: ulcerative colitis, DSS: dextran sodium sulfate, PCP: *Polygonatum cyrtonema* polysaccharide, ABX: antibiotics.

The effect of gut microbiota on the therapeutic effects of PCPs was determined by depleting gut microbiota in the UC mice using an ABX. The colon length of mice in the DSS + PCP group was significantly longer than that in the ABX + DSS + PCP group (Figures 6A,B, *p <* 0.05). Mice in the DSS + PCP, ABX + DSS, and ABX + DSS + PCP groups showed decrease in body weight. Compared with the DSS + PCP group, the ABX + DSS + PCP group showed a significantly higher degree of weight loss (Figure 6C). Compared with the ABX + DSS + PCP group, the DSS + PCP group showed a significant reduction in the DAI scores and kidney organ index (*p <* 0.05), while there were no significant changes in liver and spleen organ index (Figures 6D–G). H&E and Masson’s trichrome staining images revealed structural disruption, a reduction in goblet cells, loss of crypt architecture, an increase in inflammatory cell infiltration, and a significant increase in fibrotic area in the colon tissues of mice from the ABX + DSS + PCP group compared with the DSS + PCP group (*p <* 0.05), indicating a diminished therapeutic effect of PCPs (Figures 7A–C). The qRT-PCR results showed that the expression of *TNF-α* mRNA in colon tissues of mice was significantly increased in the ABX + DSS + PCP group compared with the DSS + PCP group (*p <* 0.01), whereas the expression of *IL-10* mRNA was significantly decreased (*p <* 0.05). However, no significant differences were found in the expression of *IL-6* and *IL-1β* mRNA among the three treatment groups (Figures 7D–G). Western blot results indicated that the level of the fibrosis-related protein α-SMA in mouse colon tissues from the DSS + PCP group was significantly lower than that in the ABX + DSS + PCP group (*p <* 0.01). However, TGF-β1 levels were not significantly different between the two groups (Figures 7H–J). Furthermore, the levels of tight junction proteins ZO-1, occludin, and claudin in the colonic mucosa of mice were significantly higher in the DSS + PCP group compared with those in the ABX + DSS + PCP group (Figures 7H, K–M, *p <* 0.01).

**FIGURE 8:**
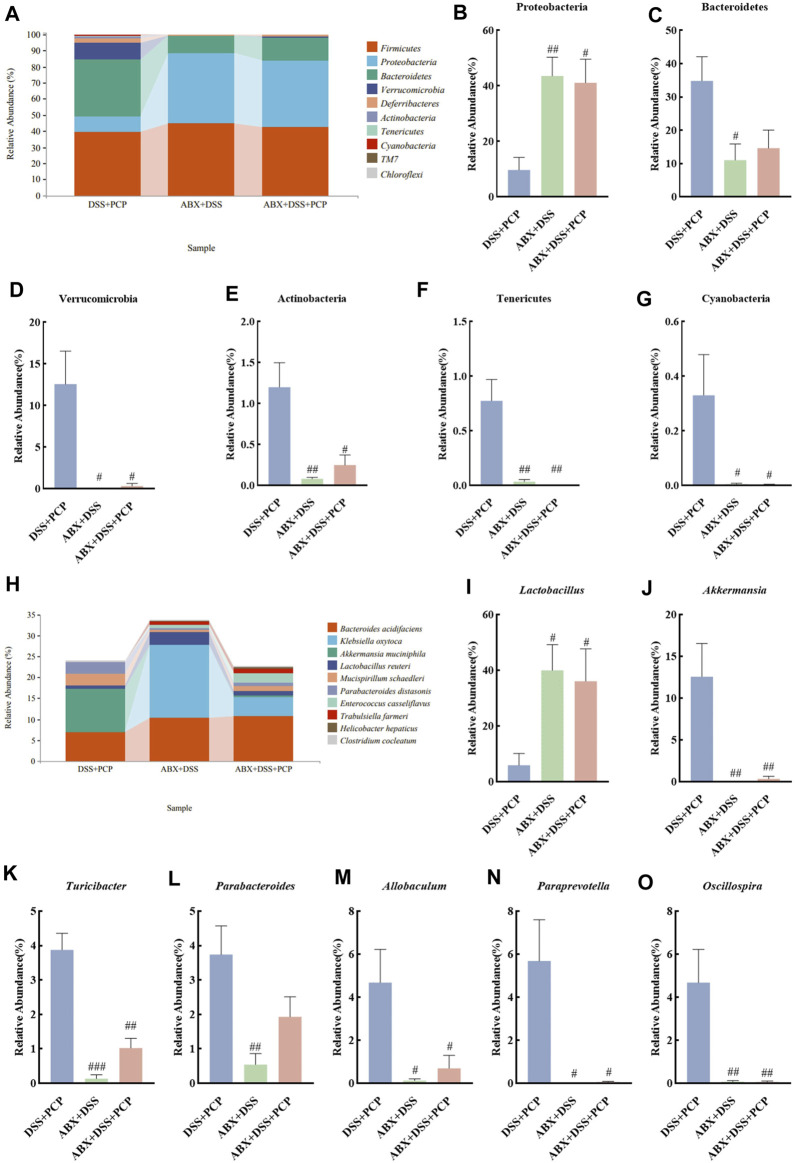
ABX treatment reverses the effect of PCPs on fecal microbiota of DSS-induced UC mice (*n* = 4/5). **(A)** Bar plot illustrates the relative abundance of the top 10 taxa at the phylum level. **(B–G)**: Differential relative abundance at the phylum level. **(H)**: Bar plot illustrates the relative abundance of the top 10 taxa at the genus level. **(I–O)**: Differential relative abundance at the genus level. DSS + PCP: DSS-exposed mice treated with PCPs, ABX + DSS: mice treated with ABX and DSS, ABX + DSS + PCP: mice treated with ABX, DSS, and PCPs. ^#^ *p <* 0.05, ^##^ *p <* 0.01, and ^###^ *p <* 0.001 vs. DSS + PCP group. UC: ulcerative colitis, DSS: dextran sodium sulfate, PCP: *Polygonatum cyrtonema* polysaccharide, ABX: antibiotics.

The bacterial 16S rRNA V3–V4 region was sequenced in mouse fecal samples and analyzed to further understand the impact of ABX treatment on the composition of gut microbiota. The species abundance did not continue to rise but remained relatively stable with the increase in sequencing depth (Supplementary Figures S8A–C), suggesting that the species detected at this sequencing depth can reflect the composition of microbiota in the samples. Compared with the DSS + PCP group, ABX treatment significantly decreased the Chao1, Simpson, Shannon, observed-species, Faith’s PD, and Pielou’s indices in the ABX + DSS + PCP group (Supplementary Figures S8D–I, *p <* 0.01). The PCoA results indicated distinct gut microbiota compositions among the DSS + PCP, ABX + DSS, and ABX + DSS + PCP groups (Supplementary Figure S8J). The relative abundance of Proteobacteria (9.72%) was significantly lower in the DSS + PCP group compared with the other two groups (*p <* 0.05) at the phylum level. The proportion of Bacteroidetes at 11.05% was significantly lower in the ABX + DSS group compared with the other two groups. Verrucomicrobia, Actinobacteria, Tenericutes, and Cyanobacteria in the DSS + PCP group accounted for 10.2%, 1.2%, 0.77%, and 0.33%, respectively, of the total microbiome, and these values were significantly higher than those in the other two groups (*p <* 0.05, Figures 8A–G). The abundance of some dominant bacterial genera in the DSS + PCP group significantly decreased after ABX treatment (*p <* 0.05). These dominant genera included *Akkermansia* (0.344%), *Turicibacter* (1.02%), *Allobaculum* (0.7%), *Paraprevotella* (0.06%), and *Oscillospira* (0.06%). The abundance of *Lactobacillus* (5.91%) significantly decreased in the DSS + PCP group compared with the other two groups (*p <* 0.05). Compared with the DSS + PCP group, *Parabacteroides* (0.54%) was significantly decreased in the ABX + DSS group (*p <* 0.01, Figures 8H–O). Overall, these findings indicated that ABX treatment noticeably diminishes the therapeutic effect of PCPs.

### 3.9 Transplantation of feces from PCP-treated mice to UC mice shows similar therapeutic effects to PCPs

**FIGURE 9:**
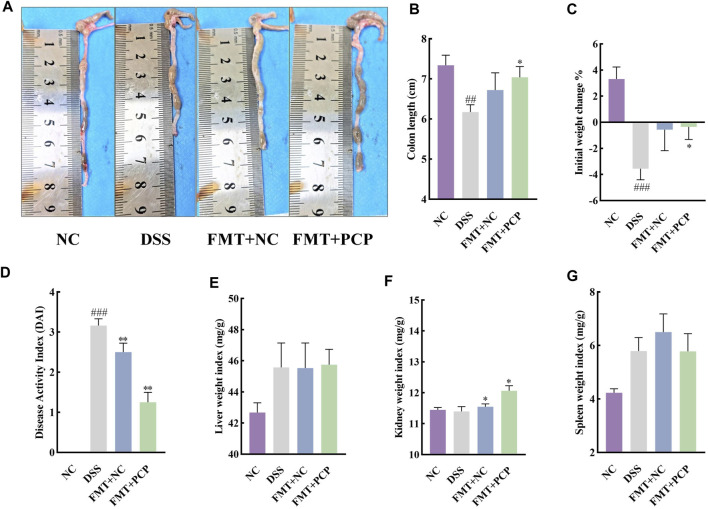
PCP treatment alleviates pathogenic phenotypic features in DSS-induced UC mice (*n* = 5/6). **(A,B)**: Quantitative analysis of colon lengths of mice in each group. **(C)**: Quantitative analysis of changes in body weights of mice in each group. **(D)**: Assessment of pathologic changes in mouse colon using the DAI scores of each group. **(E–G)**: Organ indices of the liver, kidney, and spleen from mice in each group. Data are presented as mean ± SEM. The experimental groups comprise NC (normal control mice), DSS (UC model mice), FMT + NC (NC mice receiving FMT), and FMT + PCP (PCP-treated UC mice receiving FMT). ^##^ *p <* 0.01 and ^###^ *p <* 0.001 vs. NC, ^*^ *p <* 0.05 and ^**^ *p <* 0.01 vs. DSS. UC: ulcerative colitis, DSS: dextran sodium sulfate, PCP: *Polygonatum cyrtonema* polysaccharide, FMT: fecal microbiota transplantation.

**FIGURE 10:**
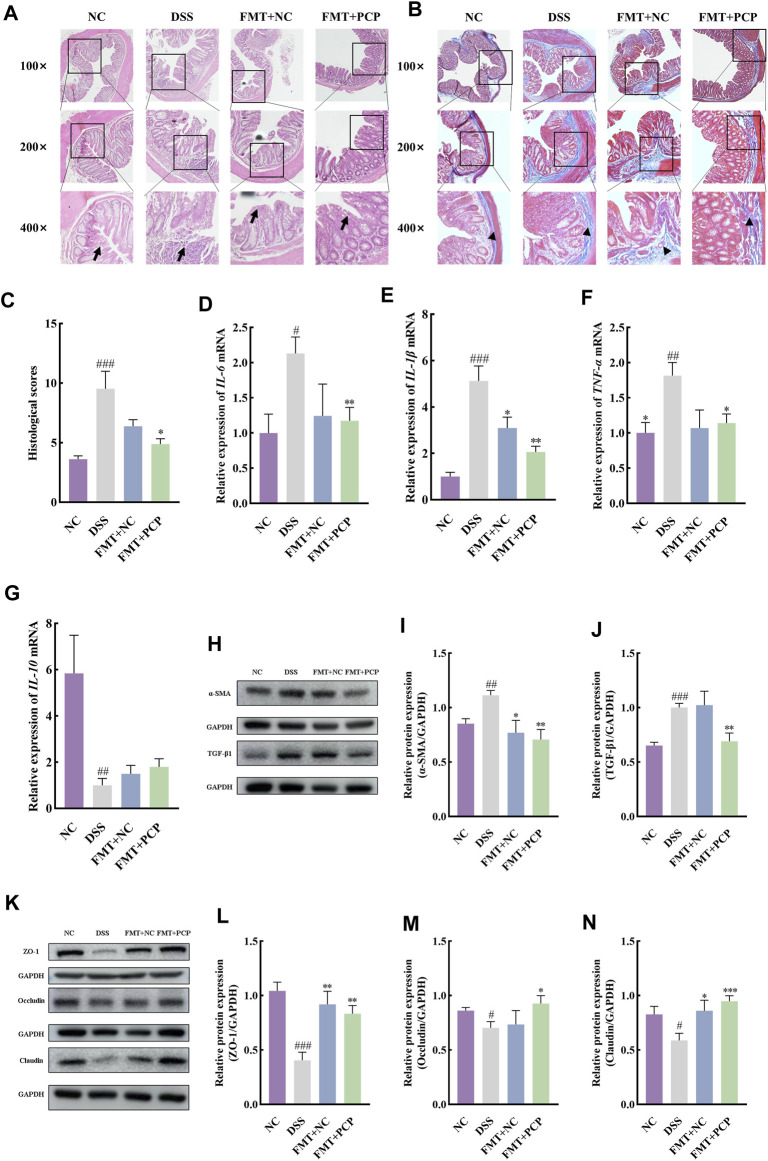
PCP treatment alleviates colon tissue damage, inflammatory imbalance, and mucosal injury in DSS-Induced UC mice (*n* = 4/5). **(A,B)**: H&E and Masson’s trichrome staining images of colon tissue sections at 100×, ×200, and ×400 magnifications show inflammatory cell infiltration (➔) and collagen deposition (▲). **(C)**: Quantification of collagen volume fraction in colon tissues using Masson’s trichrome staining. **(D–G)**: *IL-6*, *IL-1β*, *TNF-α*, and *IL-10* mRNA expression in colon tissues of mice, with *GAPDH* as the housekeeping gene. **(H–J)**: Protein levels of α-SMA and TGF-β1 in colon tissues of mice, with GAPDH as the loading control. **(K–N)**: Protein levels of ZO-1, occludin, and claudin in mouse colon tissues, with GAPDH as the loading control. Data are presented as mean ± SEM. The experimental groups comprise NC (normal control mice), DSS (UC model mice), FMT + NC (NC mice receiving FMT), and FMT + PCP (PCP-treated UC mice receiving FMT). ^#^ *p <* 0.05, ^##^ *p <* 0.01, and ^###^ *p <* 0.001 vs. NC, ^*^ *p <* 0.05, ^**^ *p <* 0.01, and ^***^ *p <* 0.001 vs. DSS. UC: ulcerative colitis, DSS: dextran sodium sulfate, PCP: *Polygonatum cyrtonema* polysaccharide, FMT: fecal microbiota transplantation.

The fecal samples from mice in the NC or PCP group were transplanted into UC mice to determine whether the therapeutic effect of PCPs was achieved by modulating gut microbiota. A significant reduction was observed in the body weight of mice in the DSS, FMT + NC, and FMT + PCP groups. However, the weight loss percentage was lower in the FMT + NC and FMT + PCP groups compared with that in the DSS group. Notably, the FMT + PCP group showed a significantly lower percentage of weight loss compared with the DSS group (Figure 9C). Furthermore, UC mice receiving fecal transplantation from the DSS + PCP group showed an increase in colon length and reduction in the DAI scores similar to the donors (*p <* 0.05). Additionally, the transplantation of fecal microbiota from mice from the NC or PCP group significantly increased the kidney weight index in UC mice (*p <* 0.05). However, the liver and spleen weight indices remained unchanged (Figures 9A,B, D–G). H&E and Masson’s trichrome staining images revealed that the therapeutic effects of the FMT + PCP treatment were similar to those of PCPs (Figures 10A–C). Moreover, the expression of *IL-6*, *IL-1β*, and *TNF-α* mRNA (Figures 10D–F) and fibrosis-related proteins α-SMA and TGF-β1 (Figures 10H–J) in colon tissues were significantly decreased in the FMT + PCP group (*p <* 0.05). The protein levels of epithelial mucosal tight junction proteins ZO-1, occludin, and claudin in the FMT + PCP group were significantly upregulated (*p <* 0.05, Figures 10K–N). However, fecal transplantation from the NC or PCP group did not significantly alter the expression of *IL-10* mRNA in mouse colon tissues (Figure 10G). Nevertheless, fecal transplantation from the PCP group had a better therapeutic effect than that from the NC group.

**FIGURE 11:**
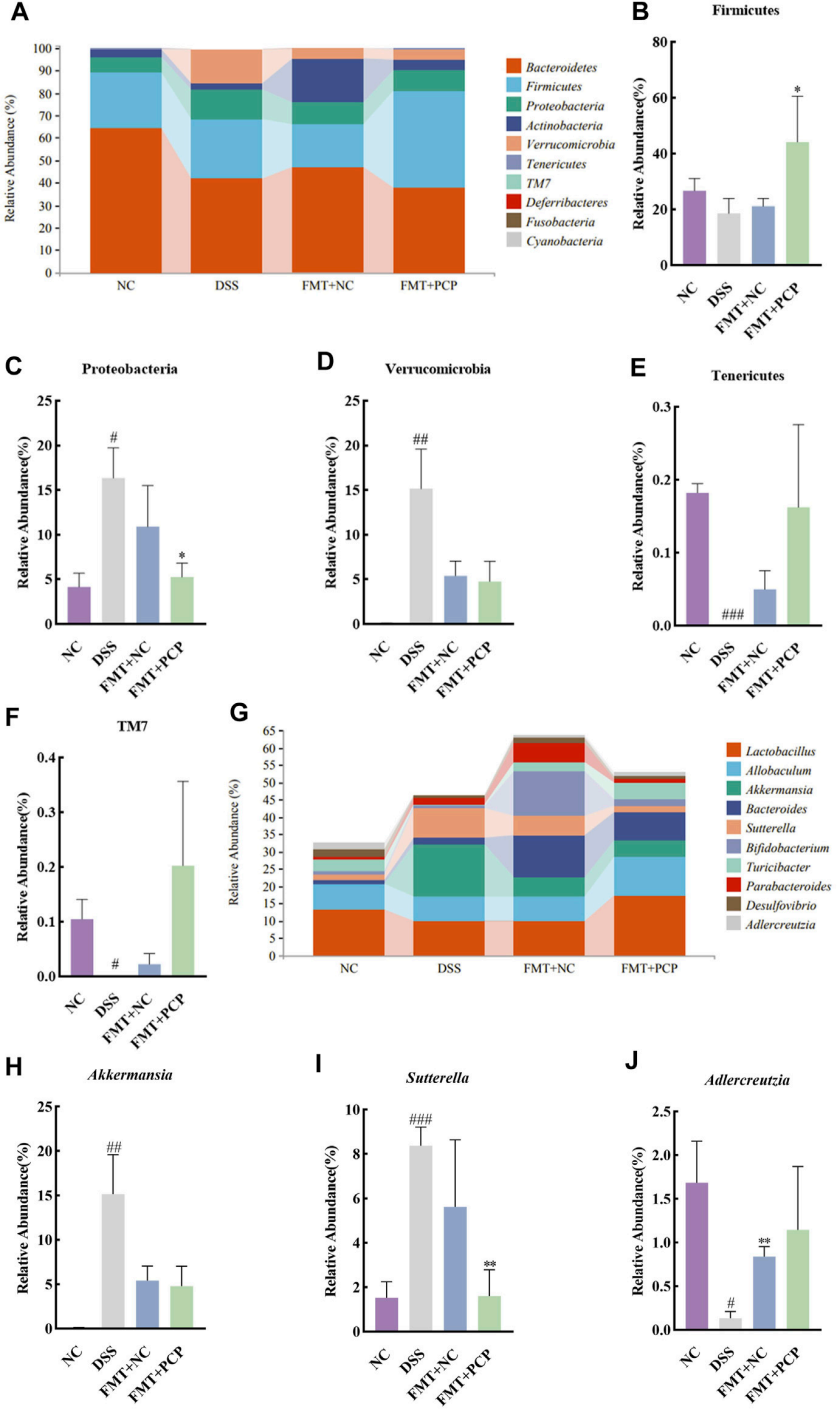
PCP-treated mice regulate the fecal microbiota composition in DSS-induced UC mice (*n* = 4). **(A)** Bar plot depicts the composition of the top 10 species at the phylum level by relative abundance. **(B–F)**: Differential relative abundance at the phylum level. **(G)** Bar plot depicts the composition of the top 10 taxa at the genus level by relative abundance. **(H–J)**: Differential relative abundance at the genus level. The experimental groups consist of NC (normal control mice), DSS (UC model mice), FMT + NC (NC mice receiving FMT), and FMT + PCP (PCP-treated UC mice receiving FMT). ^#^ *p <* 0.05, ^##^ *p <* 0.01, and ^###^ *p <* 0.001 vs. NC, ^*^ *p <* 0.05 and ^**^ *p <* 0.01 vs. DSS. UC: ulcerative colitis, DSS: dextran sodium sulfate, PCP: *Polygonatum cyrtonema* polysaccharide, FMT: fecal microbiota transplantation.

The fecal microbial community structure was determined to evaluate the effect of transplanting fecal microbiota from PCP-treated mice on the gut microbiota composition in UC mice. Species abundance did not increase but remained relatively stable with an increase in sequencing depth, indicating that the species detected at this sequencing depth could reflect the microbial composition in the samples (Supplementary Figures S9A–C). Transplantation of fecal microbiota from either the NC or PCP-treated mice did not significantly affect microbial alpha diversity indices (Supplementary Figures S9D–I). PCoA revealed differences in gut microbiota composition among the NC, DSS, FMT + NC, and FMT + PCP groups (Supplementary Figure S9J). The top 10 dominant phyla in all four groups were observed at the phylum level (Figures 11A–H). The abundance of Tenericutes and TM7 significantly decreased (*p <* 0.05), whereas that of Proteobacteria (16.37%) and Verrucomicrobia (15.16%) significantly increased (*p <* 0.05) in the DSS group compared with the NC group. The abundance of Firmicutes (44.15%) and Proteobacteria (5.26%) was restored in UC mice receiving fecal microbiota from PCP-treated mice (*p <* 0.05). However, mice treated with fecal microbiota from the NC group did not show significant changes in the gut microbiome at the phylum level (Figures 11B–F). The genus-level abundance of *Akkermansia* (15.15%) and *Sutterella* (8.37%) significantly increased (*p <* 0.01), whereas that of *Adlercreutzia* (0.13%) significantly decreased (*p <* 0.05) in the DSS group compared with the NC group. The abundance of *Adlercreutzia* (0.83%) was significantly restored in the fecal microbiota (*p <* 0.01) of mice receiving fecal microbiota from the NC group mice. The abundance of *Sutterella* (1.60%) was significantly decreased in UC mice transplanted with fecal microbiota from PCP-treated mice (*p <* 0.01, Figures 11G–J).

## 4 Discussion

UC is clinically characterized by several symptoms, including diarrhea, rectal bleeding, abdominal pain, and weight loss (Cao et al., 2022). In this study, PCPs significantly ameliorated the symptoms of weight loss, colon length shortening, increased liver and spleen weight indices, and high DAI scores in UC mice. Oxidative stress is a state of imbalance between oxidative processes and antioxidant defenses in the body, leading to neutrophil infiltration, protease secretion increase, intestinal mucosal damage, and exacerbation of UC symptoms (Wu et al., 2023). Moreover, abnormal inflammation can disrupt the anti-inflammatory system in the intestine, and chronic inflammation in the body eventually leads to colon tissue fibrosis, enhancing the progression of UC (D'Alessio et al., 2022; Shao et al., 2019). PCP treatment significantly alleviated oxidative stress in UC mice, increased the levels of the anti-inflammatory factor IL-10, and decreased the levels of pro-inflammatory factors IL-6, IL-1β, and TNF-α and fibrosis-related proteins α-SMA and TGF-β1. Furthermore, an intact and effective colonic mucosal barrier is critical to prevent the entry of harmful microbial pathogens and other antigens from the intestinal lumen. It ensures the normal absorption of nutrients such as water and sodium in the colonic mucosa, thereby preserving colonic function and preventing the leakage and formation of ulcers in the intestinal mucosa (Hao et al., 2022). In this study, PCP treatment significantly increased the expression of tight junction proteins (ZO-1, occludin, and claudin) in the colon tissues of DSS-treated mice, indicating the therapeutic effect of PCPs on UC.

The changes in intestinal microbial diversity are indicative of the severity of UC. Prior research has suggested substantial changes in the gut microbiota of UC mice (Huang et al., 2022). In this study, an increase in the abundance of *UBA3263* sp001689615, *CAG-873* sp910587235, and *UBA7173* sp013316535 was observed in the fecal microbiota of UC model mice. These species, enriched in UC model mice, have been associated with potential harm. For instance, *CAG-873* sp910587235 is a carbohydrate-active enzyme complement that can promote barrier dysfunction (Bowerman et al., 2020). The disruption of intestinal gut bacteria is closely associated with the development of UC. The abundance of *A. muciniphila* A, *Muribaculum*, and *Duncaniella* was significantly decreased in DSS-induced model mice. Notably, these species are potential probiotics. *A. muciniphila* A can alleviate metabolic abnormalities in UC mice (including fat mass increase, metabolic endotoxemia, and adipose tissue inflammation) and increase the endocannabinoids involved in regulating inflammation, intestinal barrier function, and gut peptide secretion. These effects improve intestinal barrier integrity, energy balance, and blood parameters and ameliorate inflammation (Jian et al., 2023). *Muribaculum* contributes to carbohydrate esterase (CE 14) and glycoside hydrolase (GH 37) activities and has been associated with obesity prevention (Huang Y. et al., 2023). *Duncaniella* prevents intestinal epithelial damage and alleviates symptoms associated with UC (Chang et al., 2021).

Gut microbiota is a complex microbial ecosystem, and metabolic products derived from gut microbes can protect the integrity of the structure and function of intestinal mucosa (Mills et al., 2022). Here, untargeted metabolomics and species–metabolite correlation analysis revealed significant correlations between specific species and metabolites. For example, “Antheraxanthin,” recognized for its antioxidant activity, was negatively correlated with *CAG-873* sp910587235 and *UBA7173* sp013316535 and positively correlated with *A. muciniphila* A (Shimode et al., 2018). “Biochanin A,” a natural fatty acid amide hydrolase (FAAH) inhibitor, was negatively correlated with *Alistipes* sp910577475 and *CAG−873* sp910587235 and positively correlated with *A. muciniphila* A. FAAH is a key enzyme involved in the degradation of endocannabinoids, and inactivation of FAAH is a therapeutic strategy for alleviating inflammation and pain associated with various central and peripheral nervous system diseases (Lodola et al., 2015). The differentially abundant metabolites in UC mice were significantly enriched in several metabolic pathways, including steroid hormone biosynthesis, prostate cancer, cholesterol metabolism, linoleic acid metabolism, and arachidonic acid metabolism. Steroid hormones have potent anti-inflammatory effects and can regulate cell growth, development, metabolic homeostasis, cognition, mental health, immune homeostasis, and cell apoptosis (van der Giessen et al., 2019; Han et al., 2021). Administration of PCPs partially alleviated the dysbiosis of gut microbiota and fecal metabolome.

PCP samples were fractionated to identify the biologically active fractions. Our findings revealed that unfractionated PCP showed superior therapeutic effects on UC compared with PCP-1 and PCP-2. This phenomenon can be attributed to the synergistic action of two polysaccharide fractions present in the unfractionated PCP. Furthermore, the therapeutic effects of PCPs were partially attributed to changes in the gut microbiota. The treatment with ABX significantly diminished the therapeutic effects of PCPs on UC and diminished the beneficial effects of PCPs in promoting gut microbiota balance. The abundance of *Bacteroidetes*, *Akkermansia*, *Turicibacter*, *Allobaculum*, *Paraprevotella*, and *Oscillospira* decreased in mouse feces, whereas that of *Proteobacteria* significantly increased after ABX treatment. *Turicibacter* is involved in fermentation metabolism, and lactic acid is its main metabolic product, which plays a role in regulating muscle metabolism and combating fatigue (Wang et al., 2023). *Allobaculum* and *Oscillospira* can produce short-chain fatty acids to improve gut barrier function and exert anti-inflammatory effects (Xu et al., 2022; Chen et al., 2023). *Paraprevotella* can reduce pancreatic protease concentrations in the colonic lumen, thereby protecting intestinal integrity (Li et al., 2022). *Proteobacteria* is a pathogenic phylum, and the increase in its abundance exacerbates the severity of UC in mice (Wong et al., 2008). FMT is a novel therapy for gastrointestinal and non-gastrointestinal diseases (Smits et al., 2013). However, the risks associated with FMT require long-term investigations. Our findings indicated that the effects of PCPs can be partially achieved through FMT, and FMT from PCP-treated mice had a stronger inhibitory effect on UC compared with that from NC mice. In addition, the abundance of *Akkermansia* significantly increased in the DSS-treated mice after FMT. Therefore, we hypothesized that the increase in the abundance of *Akkermansia* in the colons of UC mice during recovery repaired the DSS-induced damage to the colon tissue. However, *Akkermansia* also assumes a pro-inflammatory role in the development of inflammatory bowel disease (Wang et al., 2022). Therefore, further research is required to investigate the role of *Akkermansia* in UC.

In conclusion, PCPs mitigate the symptoms associated with DSS-induced UC by modulating the gut microbiota. However, we have not explored the precise relationship between gut microbiota and oxidative stress, inflammatory factors, colon tissue fibrosis, and tight junction protein expression. Future research efforts should focus on exploring the correlations among these factors and identifying key bacterial species in the gut microbiota that are crucial to the development of UC. These research efforts may provide more comprehensive experimental support for the development of UC therapies using PCPs as source material.

## Data Availability

The original contributions presented in the study are included in the article/Supplementary Material, further inquiries can be directed to the corresponding authors.
