# Supplementary material for: Polygonatum cyrtonema polysaccharides reshape the gut microbiota to ameliorate dextran sodium sulfate-induced ulcerative colitis in mice
Source: Front Pharmacol. 2024 Jun 5;15:1424328. doi: 10.3389/fphar.2024.1424328 (PMC11185953; doi:10.3389/fphar.2024.1424328)
Supplement: Supplementary file 1 [file DataSheet1.docx]

Supplementary Material

**
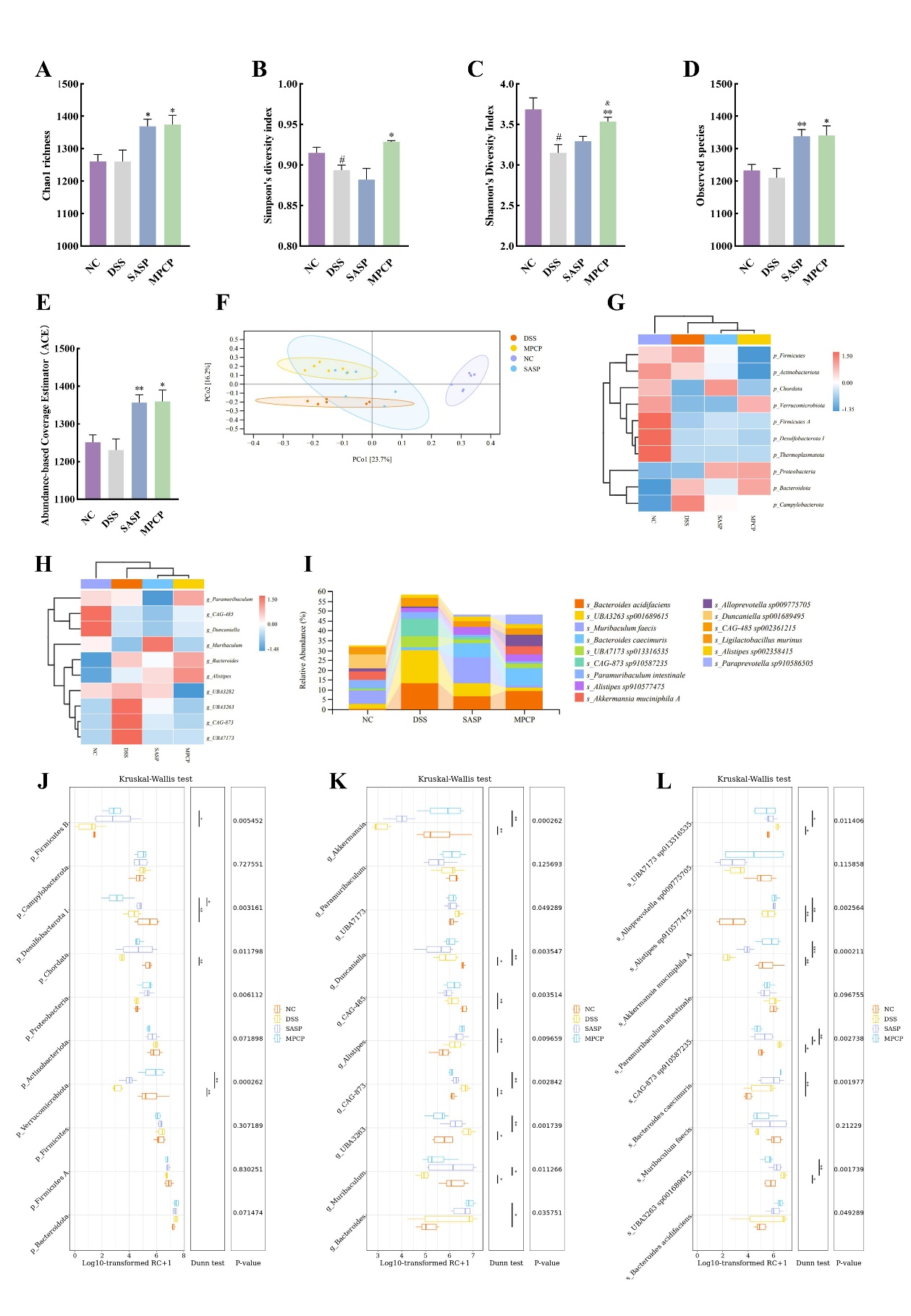
**

**Supplementary Figure 1 PCPs regulate the fecal microbiota community structure in DSS-induced UC mice (n = 6).** A–E: Alpha diversity indices, namely Chao1, Simpson, Shannon, observed-species, and ACE, for each group. F: PCoA plot illustrates the microbial community structure in each group. G–H: Heatmaps display species composition at the phylum and genus levels for each group. I: Bar plot represents the species composition for each group. J–L: Relative abundance of dominant phyla, genera, and species for each group. The experimental groups consist of NC (normal control mice), DSS (UC model mice), SASP (UC mice treated with 200 mg/kg SASP as a positive control), LPCP (UC mice treated with 40 mg/kg PCPs), MPCP (UC mice treated with 80 mg/kg PCPs), and HPCP (UC mice treated with 120 mg/kg PCPs). ^#^*p <* 0.05 vs. NC, ^*^*p <* 0.05, ^**^*p <* 0.01, and ^***^*p <* 0.001 vs. DSS, ^&^*p <* 0.05 vs. SASP. UC: ulcerative colitis, DSS: dextran sodium sulfate, PCP: *Polygonatum cyrtonema* polysaccharide, PCoA: principal coordinate analysis, SASP: sulfasalazine, LPCP: low-dose PCP, MPCP: medium-dose PCP, HPCP: high-dose PCP.


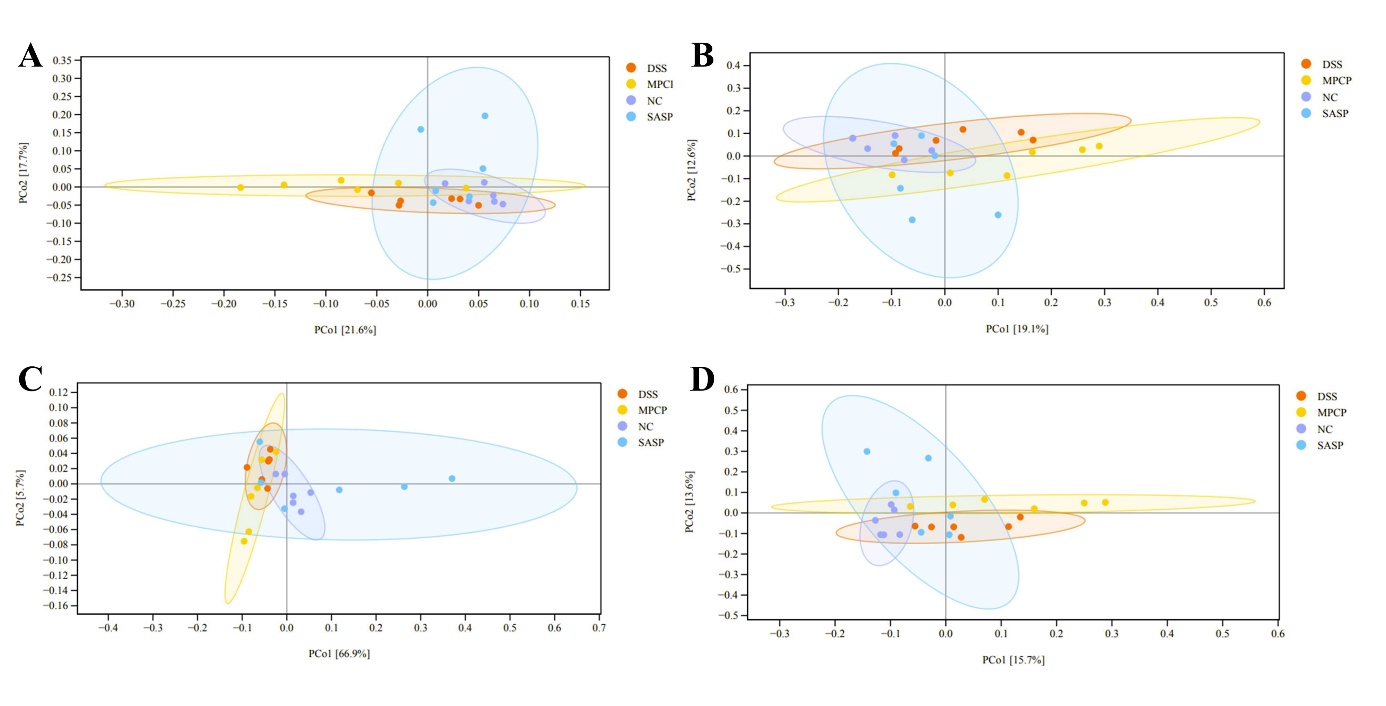


**Supplementary Figure 2 PCoA of the regulatory effect of PCPs on the functional structure of fecal microbiota in DSS-induced UC mice (n = 6).** A–D: PCoA plots illustrate the functional composition of each group in the KEGG database KO, eggNOG database OG, GO database L2, and SwissProt database. UC: ulcerative colitis, DSS: dextran sodium sulfate, PCP: *Polygonatum cyrtonema* polysaccharide, PCoA: principal coordinate analysis.


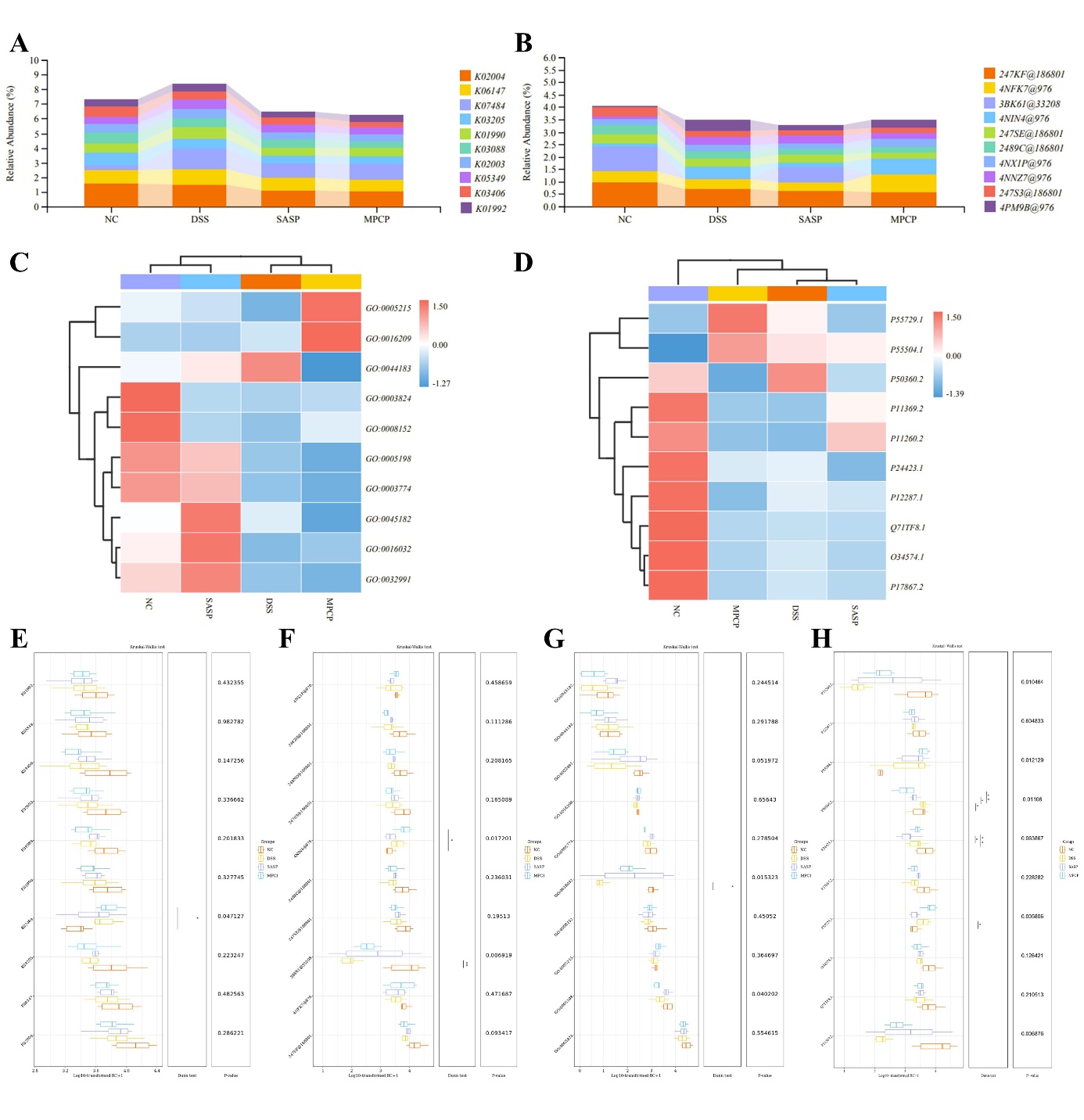


**Supplementary Figure 3 PCPs modulate the functional structure of fecal microbiota in DSS-induced UC mice (n = 6).** A–B: Bar plot depicting the functional composition in KEGG database KO and eggNOG database OG for each group. C-D: Heatmap showing the functional composition in GO database L2 and SwissProt database for each group. E–H: Differential functional analysis in KEGG database KO, eggNOG database OG, GO database L2, and SwissProt database for each group. ^*^*p <* 0.05 and ^**^*p <* 0.01. UC: ulcerative colitis, DSS: dextran sodium sulfate, PCP: *Polygonatum cyrtonema* polysaccharide.


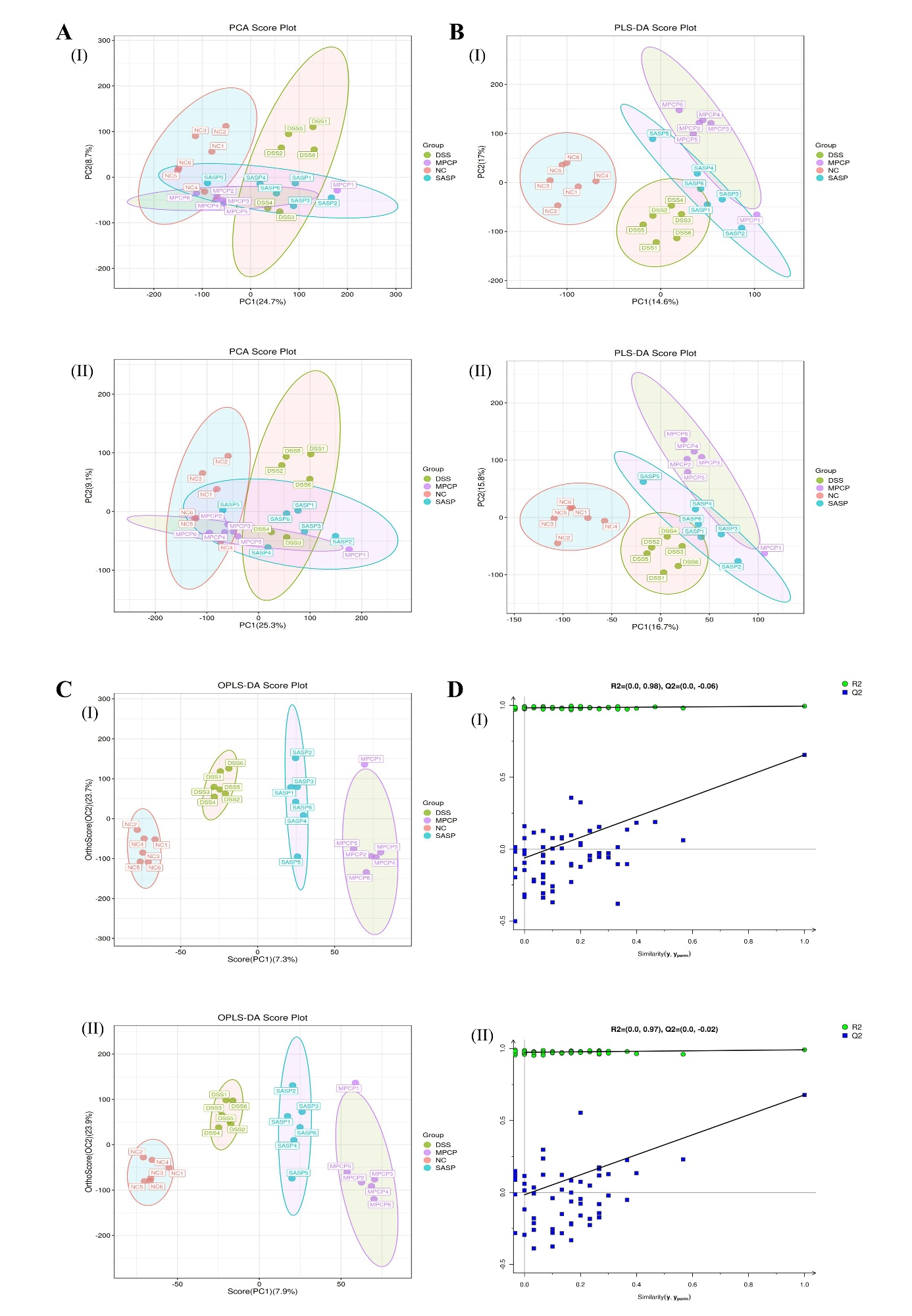


**Supplementary Figure 4 PCPs modulate metabolite multivariate analysis in DSS-induced UC mice (n = 6).** A: PCA plots (I) in positive ion (R^2^X = 0.512) and (II) negative ion (R^2^X = 0.519) modes. B: PLS–DA plots (I) in positive ion (R^2^X = 0.379, R^2^Y = 0.994, and Q^2^ = 0.9) and (II) negative ion (R^2^X = 0.389, R^2^Y = 0.991, and Q^2^ = 0.892) modes. C: OPLS–DA plots (I) in positive ion (R^2^X = 0.379, R^2^Y = 0.994, and Q^2^ = 0.655) and (II) negative ion (R^2^X = 0.389, R^2^Y = 0.991, and Q^2^ = 0.678) modes. D: OPLS–DA permutation plots (I) in positive ion and (II) negative ion modes. UC: ulcerative colitis, DSS: dextran sodium sulfate, PCP: *Polygonatum cyrtonema* polysaccharide, PCA: principal fraction analysis, PLS-DA: partial least squares-discriminant analysis, OPLS-DA: orthogonal partial least squares-discriminant analysis.


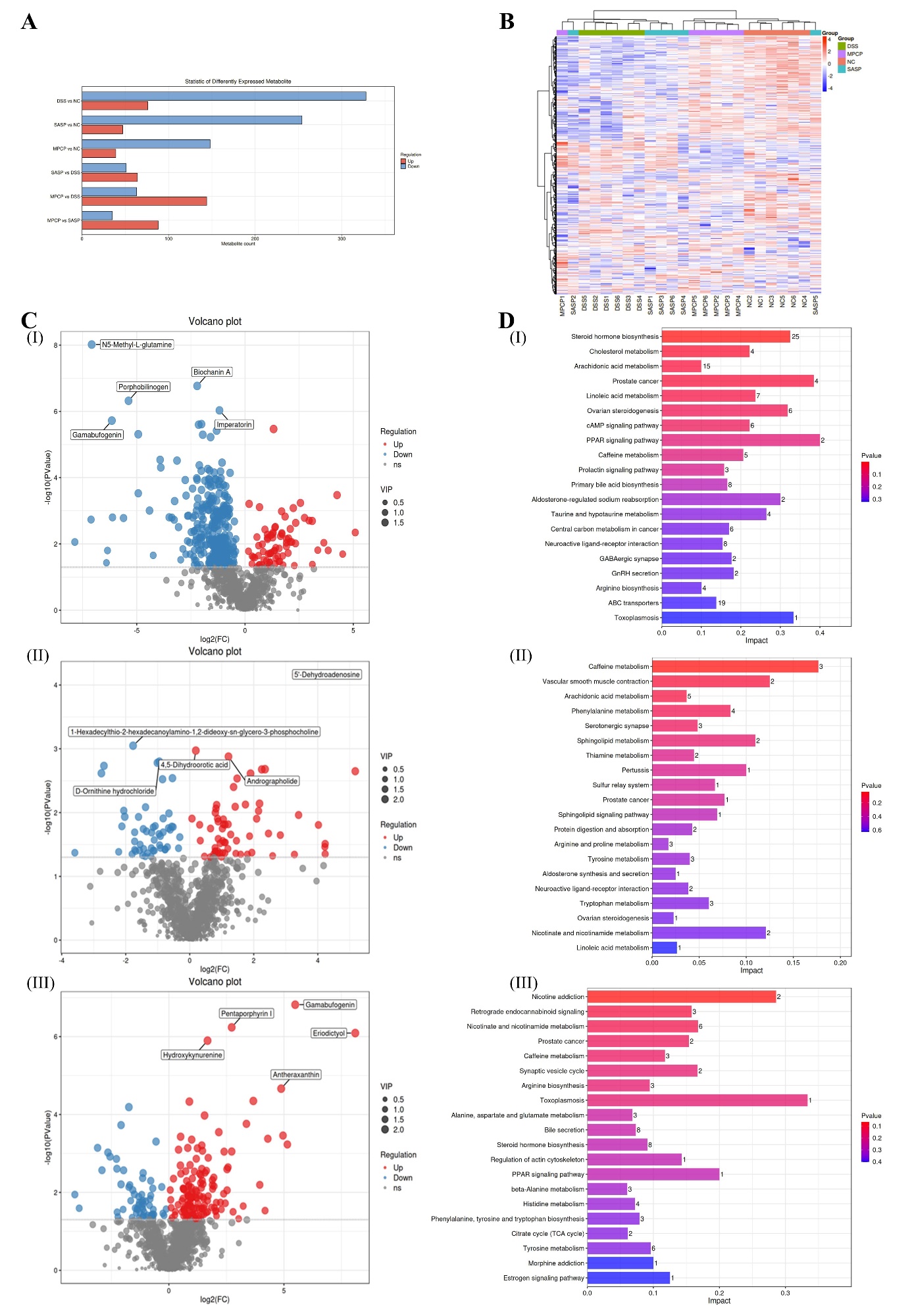


**Supplementary Figure 5 PCPs alter the fecal metabolome in DSS-induced UC mice (n = 6).** A: Number of differentially abundant metabolites in each group. B: Heatmap displaying the overall sample clustering of metabolites. C: Volcano plots of differentially abundant metabolites for (I) DSS vs. NC, (II) SASP vs. DSS, and (III) MPCP vs. DSS, with significance defined as *p* < 0.05 and VIP > 1.0. D: KEGG pathway analysis of differentially abundant metabolites for (I) DSS vs. NC, (II) SASP vs. DSS, and (III) MPCP vs. DSS. UC: ulcerative colitis, DSS: dextran sodium sulfate, PCP: *Polygonatum cyrtonema* polysaccharide, SASP: sulfasalazine, MPCP: medium-dose *Polygonatum cyrtonema* polysaccharide.


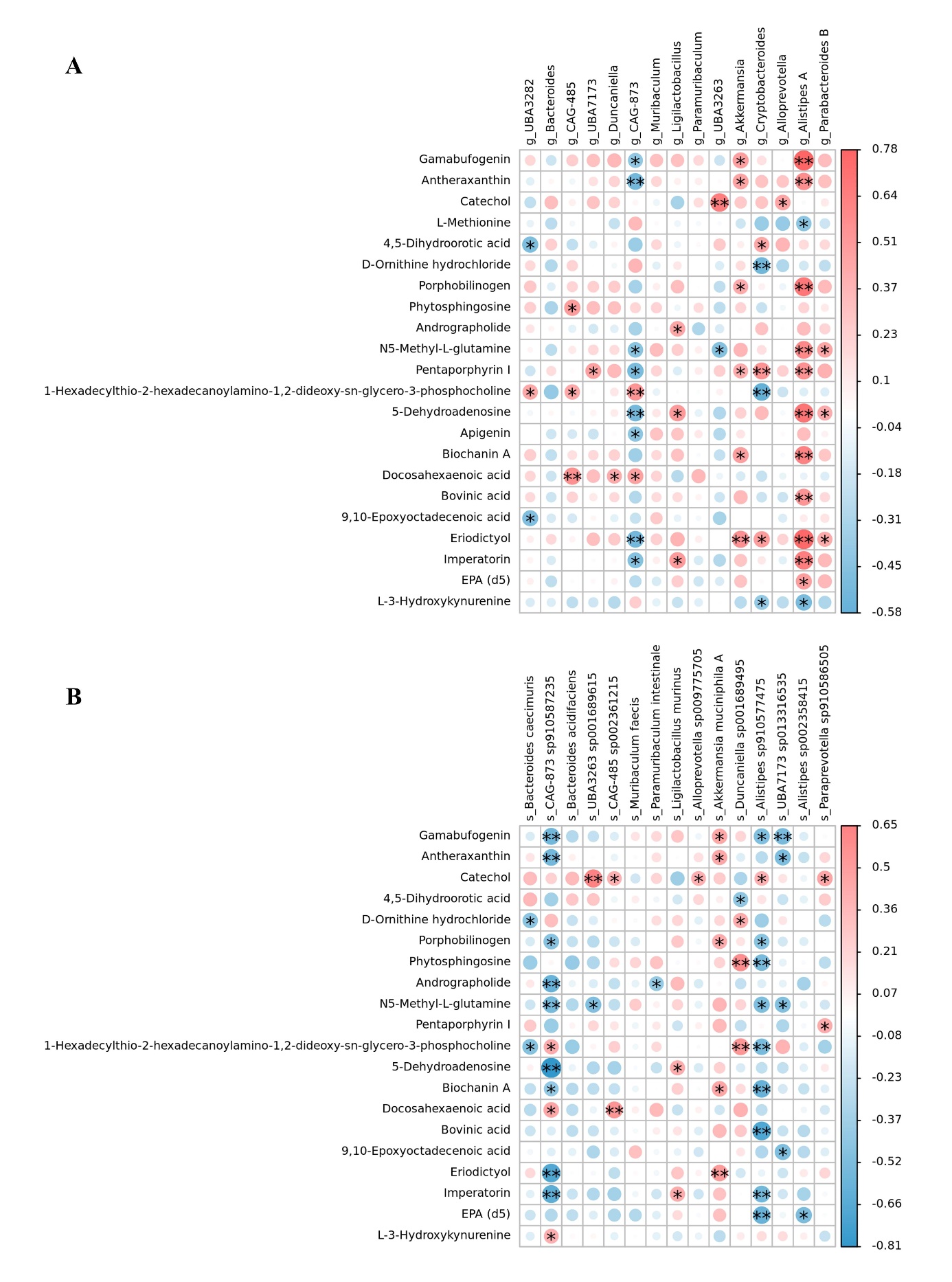


**Supplementary Figure 6 Correlation between fecal microbiota and metabolites in PCP-treated DSS-induced UC mice (n = 6).** A: Heatmap showing the correlation analysis between species at the genus level and metabolites. B: Heatmap displaying the correlation analysis between species at the species level and metabolites. ^*^ and ^**^ indicate species–metabolite correlations with *p* < 0.05 and *p* < 0.01, respectively. UC: ulcerative colitis, DSS: dextran sodium sulfate, PCP: *Polygonatum cyrtonema* polysaccharide.


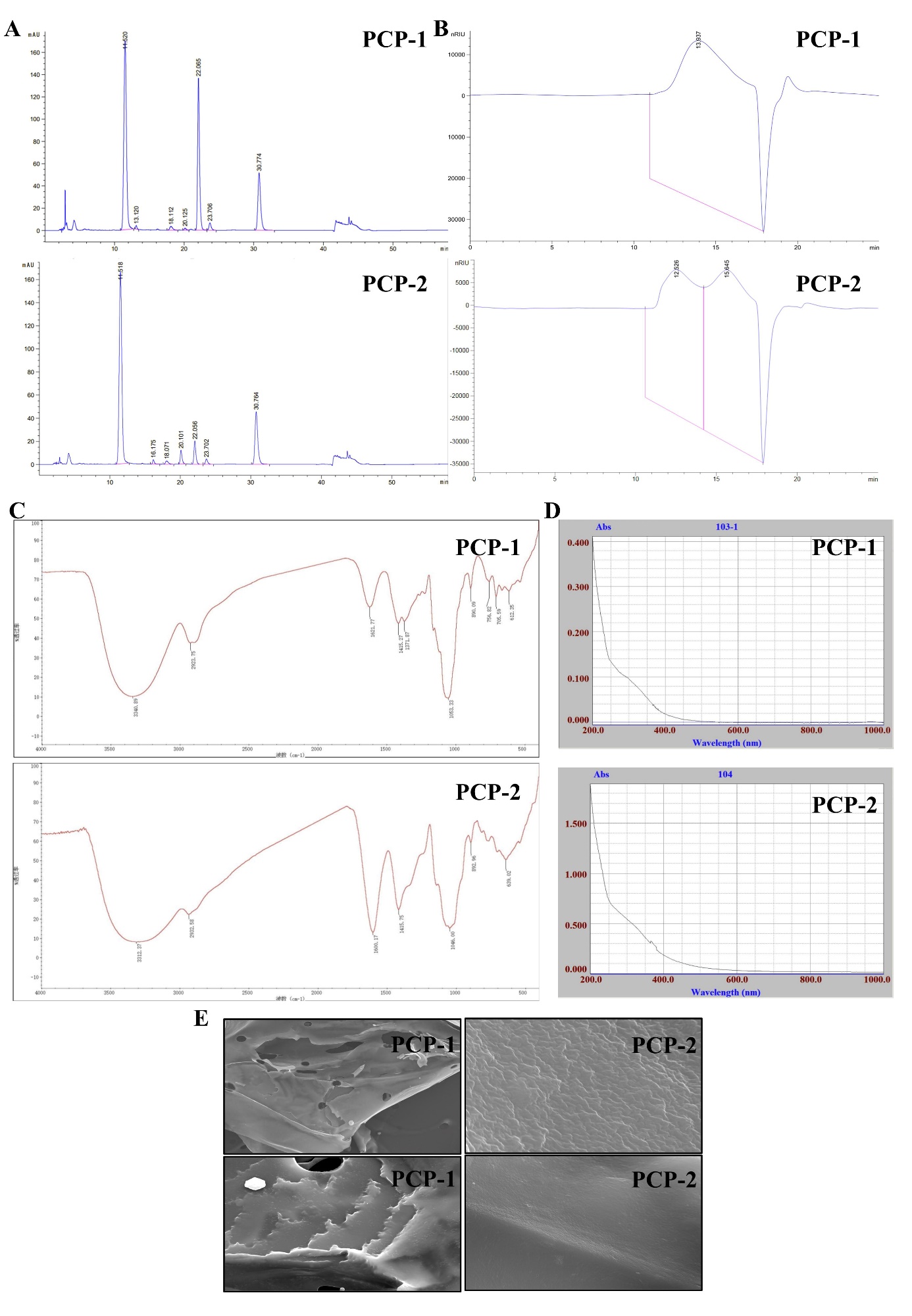


**Supplementary Figure 7 Structural characterization of PCPs.** A: Monosaccharide composition profiles. B: GPC-HPLC chromatogram. C: FT-IR spectra. D: UV spectra. E: SEM images of PCP component 1 (PCP-1) and PCP component 2 (PCP-2). UC: ulcerative colitis, DSS: dextran sodium sulfate, PCP: *Polygonatum cyrtonema* polysaccharide, ABX: antibiotics, UV: ultraviolet, GPC: gel permeation chromatography, HPLC: high-performance liquid chromatography, FT-IR: fourier-transform infrared, SEM: scanning electron microscopy.


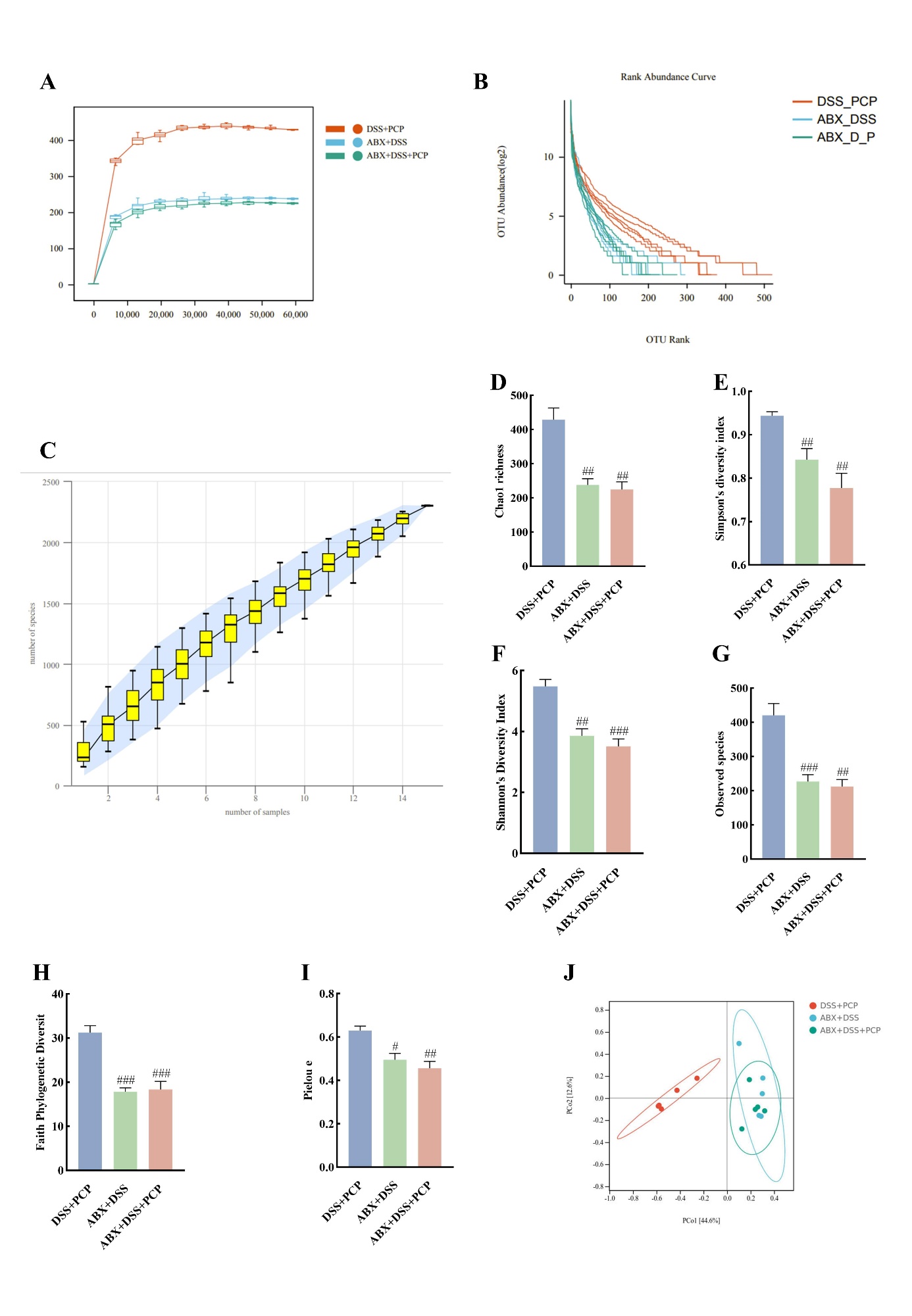


**Supplementary Figure 8 ABX treatment reverses the effects of PCPs on fecal microbiota diversity in DSS-induced UC mice (n = 5).** A: Species rarefaction curve. B: Species abundance rank curve. C: Species accumulation curve. D–I: Alpha diversity indices, namely Chao1, Simpson, Shannon, Observed species, Faith's PD, and Pielou's indices, in fecal samples from mice of each group. J: PCoA plot showing the microbial community structure in each group. DSS + PCP: DSS-exposed mice treated with PCPs, ABX + DSS: mice treated with ABX and DSS, ABX + DSS + PCP: mice treated with ABX, DSS, and PCPs. ^#^*p <* 0.05, ^##^*p <* 0.01, and ^###^*p <* 0.001 vs. DSS + PCP. UC: ulcerative colitis, DSS: dextran sodium sulfate, PCP: *Polygonatum cyrtonema* polysaccharide, ABX: antibiotics, PCoA: principal coordinate analysis.


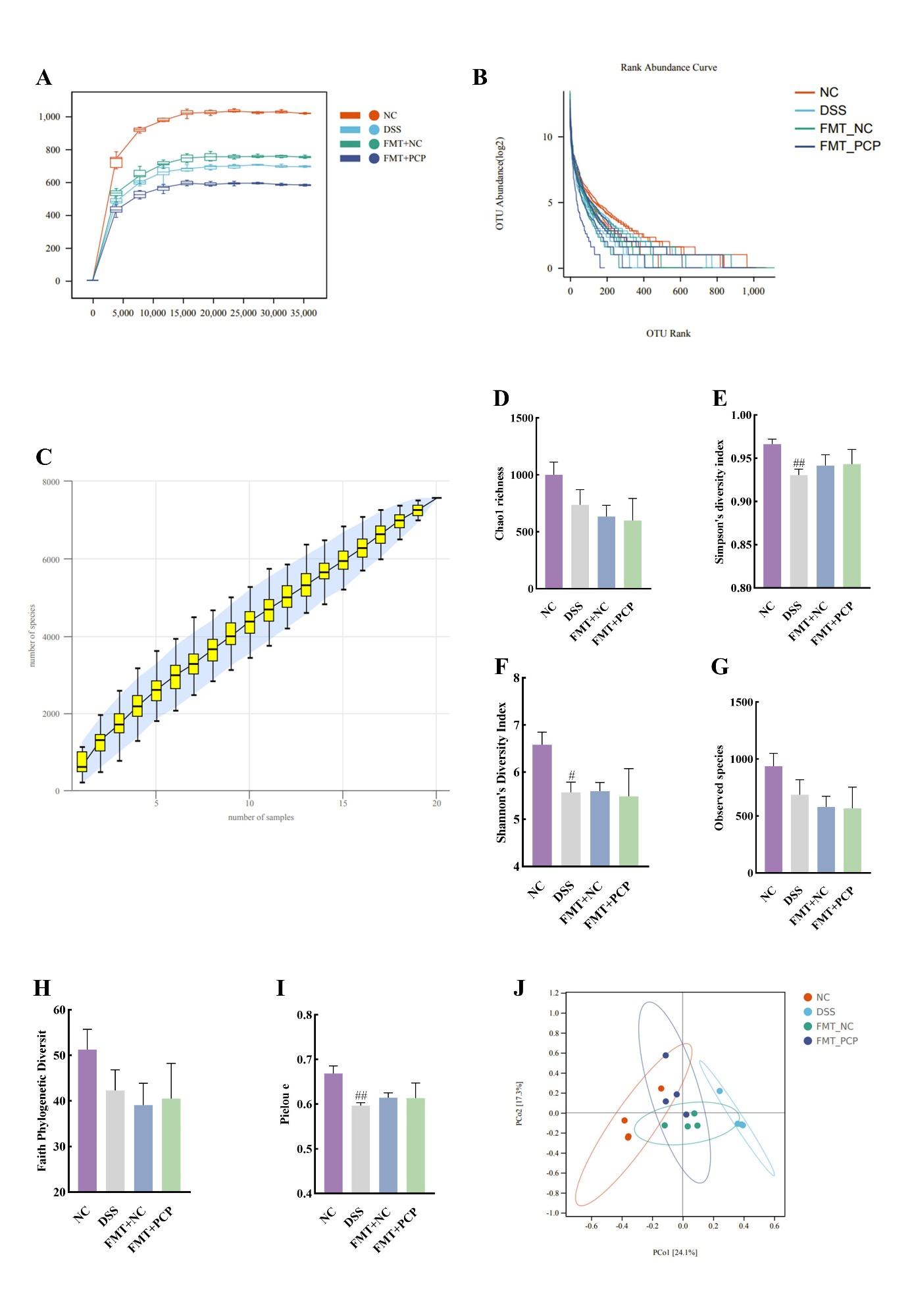


**Supplementary Figure 9 PCPs modulate fecal microbiota diversity in DSS-induced UC mice (n = 4).** A: Species rarefaction curve. B: Species abundance rank curve. C: Species accumulation curve. D–I: Alpha diversity indices, namely Chao1, Simpson, Shannon, observed species, Faith's PD, and Pielou's indices, in fecal samples from mice of each group. J: PCoA plot shows the microbial community structure in each group. The experimental groups comprise NC (normal control mice), DSS (UC model mice), FMT + NC (NC mice receiving FMT), and FMT + PCP (PCP-treated UC mice receiving FMT). ^#^*p <* 0.05 and ^##^*p <* 0.01 vs. NC. UC: ulcerative colitis, DSS: dextran sodium sulfate, PCP: *Polygonatum cyrtonema* polysaccharide, PCoA: principal coordinate analysis.
